# Regulation of p53 and Rb Links the Alternative NF-κB Pathway to EZH2 Expression and Cell Senescence

**DOI:** 10.1371/journal.pgen.1004642

**Published:** 2014-09-25

**Authors:** Alessio Iannetti, Adeline C. Ledoux, Susan J. Tudhope, Hélène Sellier, Bo Zhao, Sophia Mowla, Adam Moore, Holger Hummerich, Benjamin E. Gewurz, Simon J. Cockell, Parmjit S. Jat, Elaine Willmore, Neil D. Perkins

**Affiliations:** 1 Institute for Cell and Molecular Biosciences, Faculty of Medical Sciences, Newcastle University, Newcastle Upon Tyne, United Kingdom; 2 Northern Institute for Cancer Research, Faculty of Medical Sciences, Newcastle University, Newcastle Upon Tyne, United Kingdom; 3 Division of Infectious Disease, Brigham and Women's Hospital, Boston, Massachusetts, United States of America; 4 Department of Neurodegenerative Disease, UCL Institute of Neurology, London, United Kingdom; 5 Bioinformatics Support Unit, Faculty of Medical Sciences, Newcastle University, Newcastle Upon Tyne, United Kingdom; University of Washington, United States of America

## Abstract

There are two major pathways leading to induction of NF-κB subunits. The classical (or canonical) pathway typically leads to the induction of RelA or c-Rel containing complexes, and involves the degradation of IκBα in a manner dependent on IκB kinase (IKK) β and the IKK regulatory subunit NEMO. The alternative (or non-canonical) pathway, involves the inducible processing of p100 to p52, leading to the induction of NF-κB2(p52)/RelB containing complexes, and is dependent on IKKα and NF-κB inducing kinase (NIK). Here we demonstrate that in primary human fibroblasts, the alternative NF-κB pathway subunits NF-κB2 and RelB have multiple, but distinct, effects on the expression of key regulators of the cell cycle, reactive oxygen species (ROS) generation and protein stability. Specifically, following siRNA knockdown, quantitative PCR, western blot analyses and chromatin immunoprecipitation (ChIP) show that NF-κB2 regulates the expression of CDK4 and CDK6, while RelB, through the regulation of genes such as PSMA5 and ANAPC1, regulates the stability of p21WAF1 and the tumour suppressor p53. These combine to regulate the activity of the retinoblastoma protein, Rb, leading to induction of polycomb protein EZH2 expression. Moreover, our ChIP analysis demonstrates that EZH2 is also a direct NF-κB target gene. Microarray analysis revealed that in fibroblasts, EZH2 antagonizes a subset of p53 target genes previously associated with the senescent cell phenotype, including DEK and RacGAP1. We show that this pathway provides the major route of crosstalk between the alternative NF-κB pathway and p53, a consequence of which is to suppress cell senescence. Importantly, we find that activation of NF-κB also induces EZH2 expression in CD40L stimulated cells from Chronic Lymphocytic Leukemia patients. We therefore propose that this pathway provides a mechanism through which microenvironment induced NF-κB can inhibit tumor suppressor function and promote tumorigenesis.

## Author Summary

Although the classical NF-κB pathway is frequently associated with the induction of cellular senescence and the senescence associated secretory phenotype (SASP), the role of the alternative NF-κB pathway, which is frequently activated in hematological malignancies as well as some solid tumors, has not been defined. We therefore investigated the role of the alternative NF-κB pathway in this process. Here we report that NF-κB2 and RelB, the effectors of the alternative NF-κB pathway, suppress senescence through inhibition of p53 activity. Using primary human fibroblasts, we demonstrate that this is accomplished through NF-κB2/RelB dependent control of a previously unknown pathway, incorporating regulation of CDK4 and 6 expression as well as regulators of p21WAF1 and p53 protein stability. Loss of NF-κB2/RelB results in suppression of retinoblastoma (Rb) tumour suppressor phosphorylation, which in turn leads to inhibition of EZH2 expression and de-repression of p53 activity. Interestingly, we find that CD40 ligand stimulation of cells from Chronic Lymphocytic Leukemia patients, which strongly induces the alternative NF-κB pathway, also induces EZH2 expression. We propose that the alternative NF-κB pathway can promote tumorigenesis through suppression of p53 dependent senescence, a process that may have relevance to cancer cells retaining wild type p53.

## Introduction

In mammalian cells the NF-κB family of transcription factors consists of five subunits, RelA (p65), c-Rel, RelB, NF-κB1 (p105/p50) and NF-κB2 (p100/p52), which form a wide variety of homodimeric and heterodimeric complexes [1], [2]. In most normal, unstimulated cells, NF-κB complexes are held in an inactive form, bound to one of a family of inhibitory proteins, termed IκBs. The precursor proteins p100 and p105 can also function as IκBs, prior to their processing to p52 and p50, which function as nuclear regulatory subunits. The classical (or canonical) NF-κB pathway typically leads to the induction of RelA or c-Rel containing complexes and involves the degradation of IκBα in a manner dependent on IκB kinase (IKK) β and the IKK regulatory subunit NEMO (IKKγ). The alternative (or non-canonical) pathway, involves the inducible processing of p100 to p52, leading to the induction of p52/RelB containing complexes, and is dependent on IKKα and NF-κB inducing kinase (NIK).

Aberrantly active NF-κB is associated with many diseases, including cancer [3]. The ability to both respond to and induce inflammatory stimuli is an important component of NF-κB's role in disease [3]. NF-κB also has other functions and can contribute to tumorigenesis through inducing proliferation, metastasis as well as resistance to apoptosis [4]. However, NF-κB can also exhibit apparently contradictory functions, more akin to those of a tumor suppressor. These include pro-apoptotic activity in response to some stimuli and induction of cellular senescence [4], [5]. NF-κB is also associated with the senescence associated secretory phenotype (SASP), which can exhibit tumor promoting properties but also contribute to the effectiveness of cancer therapy [6], [7]. Other studies have suggested a role for NF-κB in protecting against senescence [8]. One common feature of these studies has been a focus on the classical branch of the NF-κB pathway and any role for the alternative NF-κB pathway has not generally been considered or functionally analyzed.

A possible explanation for apparently contradictory functions of NF-κB lies in the ‘tumor suppressor status’ of the cell. A number of studies have shown that tumor suppressors can modulate NF-κB activity and function [4]. The most studied example of tumor suppressor crosstalk with NF-κB involves p53 [2], [4], [9]–[11]. NF-κB and p53 can both be activated by many of the same stimuli with a common link frequently being DNA damaging agents, which include reactive oxygen species (ROS) [2], [9]–[12]. Crosstalk between these factors can take many forms, with reports indicating both antagonistic and co-operative behavior. Although often appearing contradictory, one conclusion of these studies is that p53 and NF-κB can modulate each-others activity, and consequently cell survival, but that the exact outcome is dependent upon the cell context. As many of these studies have been performed in cancer cell lines exhibiting a variety of genetic backgrounds, with a range of different stimuli, variability in the nature and outcome of any crosstalk might be expected. We have previously identified crosstalk between the alternative NF-κB pathway subunit p52 and p53, involving p53 modulation of p52 homodimer transcriptional activity, by inducing a change from p52/Bcl3 to p52/HDAC complexes, in addition to direct recruitment of p52 to p53 target gene promoters [13], [14]. However, there remain many unanswered questions, including how the effectors of the alternative NF-κB pathway, p52 and RelB can affect p53 dependent senescence. Moreover, whether there also exists crosstalk between these NF-κB proteins and another important tumor suppressor, the retinoblastoma gene product, Rb, is largely unexplored.

Genes whose promoters and enhancers are regulated by both p53 and NF-κB have the potential to act as ‘nodes of integration’ between these pathways. That is, they form a route through which both factors come together to influence cell fate. A number of such genes have been identified, including DR5 and Caspase 10 [15], [16]. We were interested in identifying genes encoding chromatin remodellers and transcriptional co-regulators that exhibited co-operative or antagonistic regulation by NF-κB and p53 as these have the potential to re-program the transcriptional ‘landscape’ of the cell [17]. A candidate gene that fitted this category was the Polycomb protein enhancer of zeste homolog 2 (EZH2), a histone H3 K27 methylase and component of the PRC2 complex, which previously has been shown to be repressed by p53 [18]. p53 repression of EZH2 expression is thought to be indirect, resulting from transcriptional upregulation of p21^WAF1^ expression [18]. This in turn leads to Rb mediated repression of E2F activity, a key transcription factor driving EZH2 expression [19].

EZH2 is a tumor promoter and is found over-expressed or mutated in many solid tumors and hematological malignancies [20]. A component of its ability to drive tumorigenesis derives from its ability to suppress cellular senescence [21]–[23]. This is achieved, in part, through EZH2 repression of the CDKN2A locus [21], [24], encoding the CDK inhibitor p16^Ink4a^ and the tumor suppressor p14^ARF^, which can induce p53 activity through binding its inhibitor Mdm2. p16^Ink4a^ and p14^ARF^ are both important regulators of cell senescence [25]. EZH2 has also been previously linked to NF-κB activity, where in ER negative breast cancer it can function as a coactivator, independent of its methylase activity, for both RelA and RelB [26].

In this report, we define a regulatory network through which the alternative NF-κB pathway regulates Rb activity and consequently EZH2 expression. We demonstrate that EZH2 functions as a critical ‘node’ of crosstalk between NF-κB and p53 that controls a gene regulatory network through which p52 and RelB act to suppress p53 and Rb mediated cellular senescence.

## Results

### Human dermal fibroblasts grown under normoxic conditions contain a basal level of NF-κB2 and p53 activity

**Figure 1:**
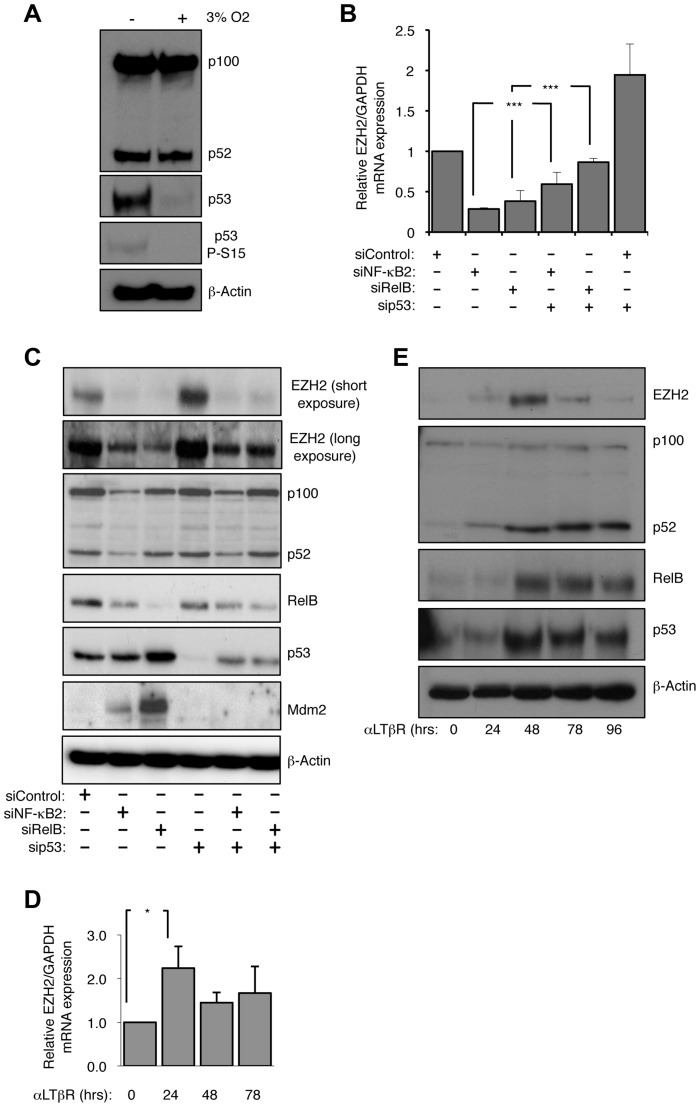
EZH2 is an NF-κB regulated target gene. (A) The basal level of p53 protein in NHD fibroblasts is ROS dependent. NHD fibroblasts were grown under normoxia at 3% O2 for 7 days before western blot analysis. (B & C) siRNA mediated knock-down of NF-κB2 and RelB leads to a reduction in EZH2 mRNA and protein levels. RNA (B) or protein (C) was prepared from NHD fibroblasts treated with the indicated siRNAs 48 hours after transfection and Q-PCR or western blot analysis was performed to determine EZH2 expression. *** P≤0.001. (D & E) Lymphotoxin β receptor stimulation leads to induction of EZH2 expression. NHD fibroblasts were treated with LTβR agonist antibody for the times indicated and either Q-PCR (D) or western blot analysis (E) was performed to determine the expression of EZH2 (D) or EZH2, p52/p100, RelB and p53 (E). * P≤0.05.

To investigate the function of the alternative NF-κB pathway in untransformed cells, we analyzed its expression in non-immortalized, normal human dermal (NHD) fibroblasts, cultured for a limited number of passages. This revealed constitutive processing of the p100 NF-κB subunit to p52 as well as a basal level of the p53 tumor suppressor (Fig. 1A). To determine if these resulted from oxidative stress due to normoxic culture conditions, the NHD fibroblasts were grown under low oxygen tension (3% O2 or treated with the antioxidant epigallocatechin-3-gallate (ECGC). This resulted in loss of p53 while processing of p100 to p52 was unaffected, suggesting the latter effect results from factors present in the media (Fig. 1A & S1A). The Ataxia Telangiectasia Mutated (ATM) kinase can be activated by ROS, independently of DNA damage [27] and consistent with this we found that the basal levels of p53 seen in this experiment as well as inducible levels seen in later experiments were reduced by treatment with an ATM kinase inhibitor (Fig. S1B). Interestingly we observed that 7 days after treatment of these cells with hydrogen peroxide (H_2_O_2_) to induce cellular senescence, a significant reduction in the processing of p100 to p52 occurred, concomitant with activation of p53 suggesting a possible antagonistic relationship between these factors in these cells (Figs. S1C&D).

### Depletion of NF-κB2 or RelB results in repression of EZH2 expression

We decided to exploit the observation that NHD fibroblasts exhibited activation of both the alternative NF-κB pathway and p53 to determine how these pathways might be integrated in a non-cancerous, non-immortalized cellular context. Consequently, we used siRNAs to deplete p53 and the alternative NF-κB pathway subunits p52/p100 (encoded by the NFKB2 gene) and RelB in the NHD fibroblasts. siRNA knockdowns of p52/p100 expression will henceforth be referred to as NF-κB2, while analysis of the individual proteins will refer to either p52 or p100.

As described above, a candidate target for NF-κB/p53 crosstalk was EZH2, a histone H3 K27 methylase and component of the PRC2 complex, previously shown to be repressed by p53 [18]. As expected siRNA knockdown of p53 resulted in an increase in EZH2 RNA and protein levels 48 hours after transfection (Fig. 1B&C). However, siRNA depletion of both alternative NF-κB pathway subunits and the p52 coactivator Bcl3 had the opposite effect, leading to almost complete loss of EZH2 expression (Fig. 1B&C, Fig. S1E–G). Knockdown of NF-κB2/RelB or Bcl3 with p53 resulted in a partial rescue of EZH2 protein levels. These effects were also seen with an EZH2 promoter luciferase construct, where loss of NF-κB2/RelB resulted in less promoter activity, while depletion of p53 had a strong stimulatory effect (Fig. S1H). As reported previously [18], depletion of the p53 target, the CDK inhibitor p21^WAF1^, also induced EZH2 promoter activity (Fig. S1H). These data suggested that the alternative NF-κB pathway and p53 antagonistically regulate EZH2 expression and that this is mediated, at least in part, through direct effects on EZH2 transcription driven by its promoter.

We next determined if induction of the alternative NF-κB pathway by a physiological stimulus would also regulate EZH2 expression. To achieve this NHD fibroblasts were treated with a lymphotoxin β receptor (LTβR) agonist antibody. Consistent with the results obtained with basal level activity of the alternative pathway, LTβR activation resulted in increased levels of EZH2 protein and mRNA (Fig. 1E & F).

In many experiments, with different siRNAs, we noted a partial depletion of RelB levels seen upon NF-κB2 knockdown. However, as there is no effect of NF-κB2 siRNAs on RelB mRNA levels and vice versa (Fig. S1F) this probably represents RelB protein instability due to an inability to form homodimers, with the remaining RelB dimerized to p50 with which it forms an active complex [28].

### EZH2 expression is induced upon CD40L stimulation of primary B-cell chronic lymphocytic leukemia (CLL) cells

**Figure 2:**
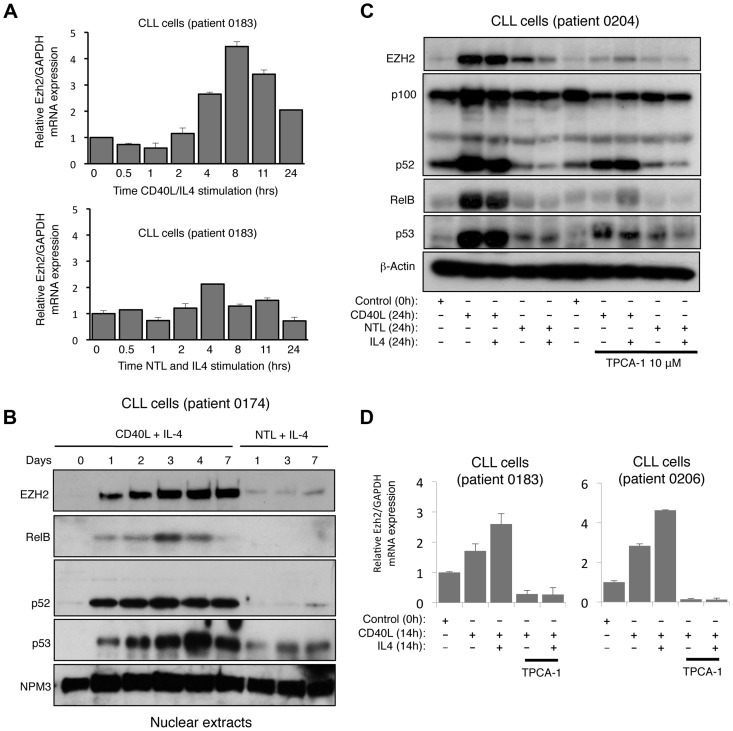
CD40 stimulation leads to NF-κB activation and CLL induction in Chronic Lymphocytic Leukemia cells. (A) Analysis of EZH2 mRNA expression in CLL cells. RNA was prepared from CLL cells stimulated with CD40L/IL4 expressing mouse fibroblasts or with untransfected fibroblasts (NTL) and IL4 for the indicated times and Q-PCR analysis of EZH2 expression was performed. (B) Analysis of EZH2 protein level in CLL cells. Western blot analysis of nuclear extracts from CLL cells stimulated with CD40L/IL4 or untransfected fibroblasts (NTL) and IL4 for the indicated times. (C & D) EZH2 protein and RNA levels in CLL cells is NF-κB dependent. Whole cell protein lysates (C) and RNA (D) were prepared from CLL cells stimulated for 24 hours with CD40L/IL4 and treated with the IKKβ inhibitor TPCA-1 where indicated.

To extend our observation that LTβR stimulation induced EZH2 expression, we were interested in whether this pathway was also associated with activation of the alternative NF-κB pathway in a pathological setting. Primary chronic lymphocytic leukemia (CLL) cells from patients can be cultured in vitro and induced to proliferate when stimulated with CD40 ligand (CD40L), which induces both the classical and alternative NF-κB pathways [29], [30] (Fig. S2A). Significantly, we observed a CD40L dependent increase in EZH2 mRNA and protein levels that was seen up to 7 days after plating (Fig. 2A&B). The reproducibility of this effect between patients was confirmed with analysis of 4 different isolates (Fig. S2B). Western blot analysis confirmed activation of the alternative NF-κB pathway, with both an increase in nuclear and overall levels of NF-κB2 and RelB being observed (Fig. 2B & S2B). The latter likely results from activation of the classical pathway by CD40L, which can ‘prime’ the alternative NF-κB pathway through inducing NF-κB2 and RelB expression levels [1]. We exploited this characteristic to confirm the role of NF-κB in EZH2 induction in CLL cells: treatment with the IKKβ inhibitor TPCA-1 efficiently blocked the induction of NF-κB2/RelB protein and mRNA levels and also abolished induction of EZH2 (Fig. 2C & D).

In addition to further confirmation of EZH2 regulation by a physiological inducer of the alternative NF-κB pathway this data also demonstrated that this pathway is not restricted to fibroblasts. Interestingly, we also observed that in most patient cells (except 0205 where p53 appears to be mutant), CD40L stimulation also induced p53 protein levels (Fig. 2A & B, S2B), an effect also seen with LTβR stimulation (Fig. 1E). As these CLL cells are being induced to proliferate (Fig. S2A) this suggests ongoing suppression/modulation of p53 activity and function.

### NF-κB2 and RelB suppress p53 dependent senescence

**Figure 3:**
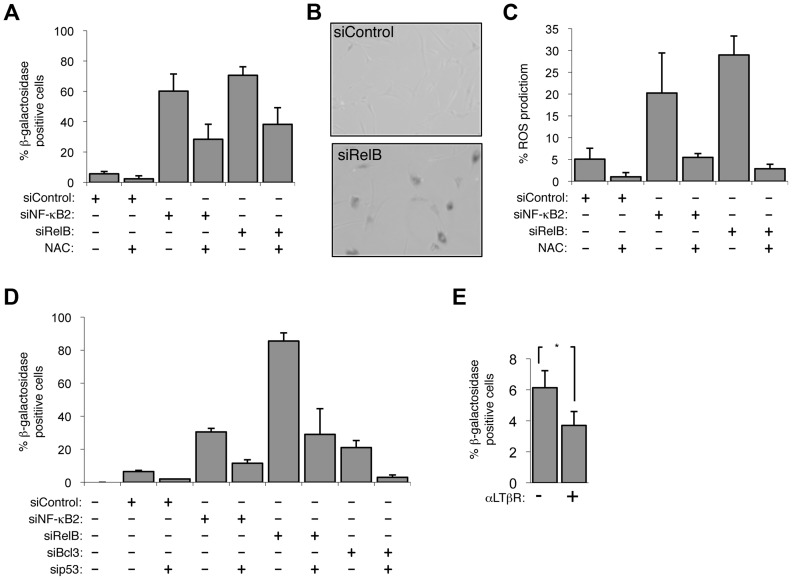
The alternative NF-κB pathway suppresses p53 mediated senescence in primary fibroblasts. (A & B) Senescence induced by siRNA knock down of NF-κB2 and RelB is ROS dependent. NHD fibroblasts were transfected with the siRNAs shown and treated, where indicated, 2 days later with the anti-oxidant N-Acetyl cysteine (NAC). 7 days after transfection cells analyzed for senescence by acidic β-galactosidase staining (A). An image of RelB siRNA transfected cells after staining is shown (B). (C) siRNA mediated knock down of NF-κB2 and RelB induces ROS production. NHD fibroblasts were transfected with the siRNAs shown and treated, where indicated, 2 days later with NAC. After 7 days they were incubated for 30 minutes with 5 mM DCF-DA and analyzed by FACs. The percentage of cells with higher than baseline ROS levels are shown. (D) siRNA knock down of NF-κB2, RelB and Bcl3 induce cellular senescence in a p53 dependent manner. NHD fibroblasts were transfected with the listed siRNAs and analyzed for senescence by β-galactosidase staining after 7 days. (E) Lymphotoxin β receptor stimulation represses basal level senescence in fibroblasts. NHD fibroblasts were treated with LTβR agonist antibody and after 7 days analyzed for senescence by β-galactosidase staining. * P≤0.05.

When analyzing siRNA depletion of NF-κB2 and RelB in NHD fibroblasts, we also observed that cells ceased to proliferate, changed morphology and after 7 days in culture, using the acidic β galactosidase assay, were found to enter a senescent state (Fig. 3A&B). This effect was confirmed with different NF-κB2 and RelB siRNAs (Fig. S3A). These effects were also associated with induction of reaction oxygen species (ROS) and loss of Lamin B1, both markers of senescence [12], [31] (Fig. 3C, Fig. S3B). Treatment of cells with the antioxidant N-acetyl cysteine (NAC) prevented senescence induced upon NF-κB2 and RelB depletion, an effect also seen with an ATM inhibitor, or ECGC (Fig. 3A, Fig. S3C–E). By contrast, depletion of the NF-κB1 (p50/p105) subunit had no detectable effect on senescence in this system (Fig. S3F). Importantly induction of senescence and ROS upon depletion of NF-κB2, RelB and Bcl-3 was p53 dependent (Fig. 3D, S3G). p53 dependent senescence was also observed upon depletion of the p52 coactivator Bcl3 (Fig. 3D). Activation of the alternative NF-κB pathway through LTβR stimulation also inhibited the basal level of senescence in NHD fibroblasts (Fig. 3E). Therefore we concluded that in NHD fibroblasts, the basal level of p53 activity induced by oxidative stress is suppressed by the alternative NF-κB pathway. Depletion of either of 3 components of this pathway, results in loss of this suppression leading to p53 dependent production of ROS and senescence.

### EZH2 is a major effector of alternative NF-κB pathway transcriptional effects

**Figure 4:**
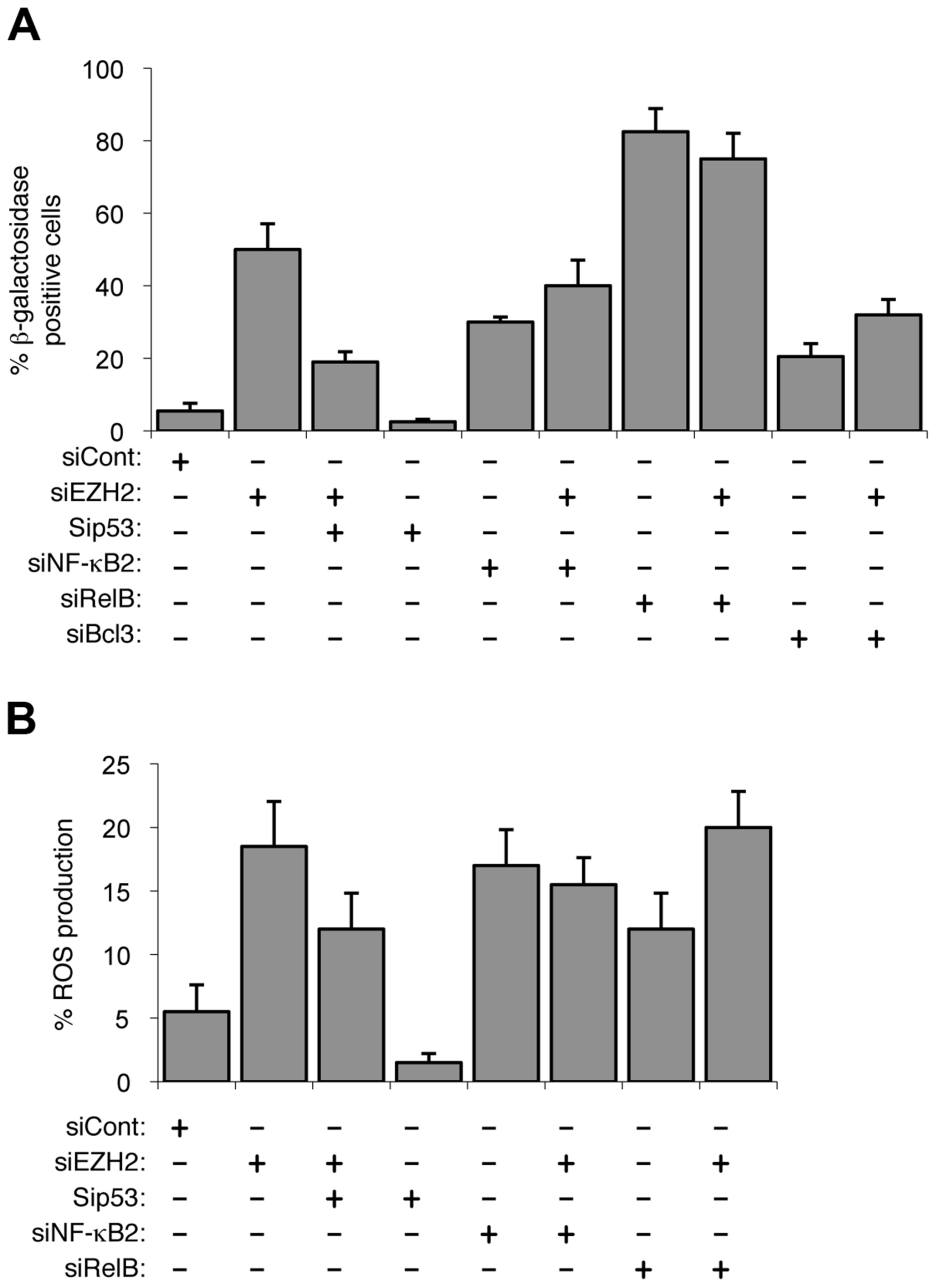
EZH2 siRNA associated senescence and ROS production is p53 dependent. (A & B) NHD fibroblasts were transfected with the siRNAs shown and analyzed for senescence (A) and ROS production (B) after 7 days.

We next examined the effect of depleting EZH2 itself. Since EZH2 can act to suppress senescence [21]–[23] we were interested in whether regulation of its expression provided a mechanism through which the alternative NF-κB pathway and p53 could exert antagonistic effects on senescence. Consistent with this hypothesis we observed that cells treated with an EZH2 siRNA became senescent and also showed elevated levels of ROS (Fig. 4A & B, S4A). Co-depletion of EZH2 with NF-κB2, RelB, Bcl-3 and p53 revealed that while EZH2 associated ROS production and senescence is p53 dependent, no additional effects were seen with the NF-κB subunits (Fig. 4A & B). This is consistent with EZH2 being a downstream ‘effector’ and master regulator of NF-κB2/RelB's ability to suppress p53 dependent senescence.

EZH2 represses expression from the CDKN2A locus encoding the CDK inhibitor p16^Ink4a^ and the tumor suppressor p14^ARF^, both of which are important regulators of senescence [21], [24]. Interestingly, 48 hours after siRNA transfection, when we analyzed effects on gene expression, no changes in p16^Ink4a^ or p14^ARF^ protein or mRNA levels could be observed (Fig. S4B & C). However, 7 days after transfection, when cells begin to senesce, this had changed with induction of the mRNAs for both factors being seen (Fig. S4C). Moreover, siRNA depletion of p14^ARF^ activity confirmed its requirement for NF-κB2, RelB and EZH2 induced senescence (Fig. S4D). Therefore, while these proteins are required, as expected, for eventual induction of the senescent phenotype, they did not seem to be directly involved in the early regulatory events linking NF-κB2, RelB to EZH2 and p53 function that are the focus of this study.

**Figure 5:**
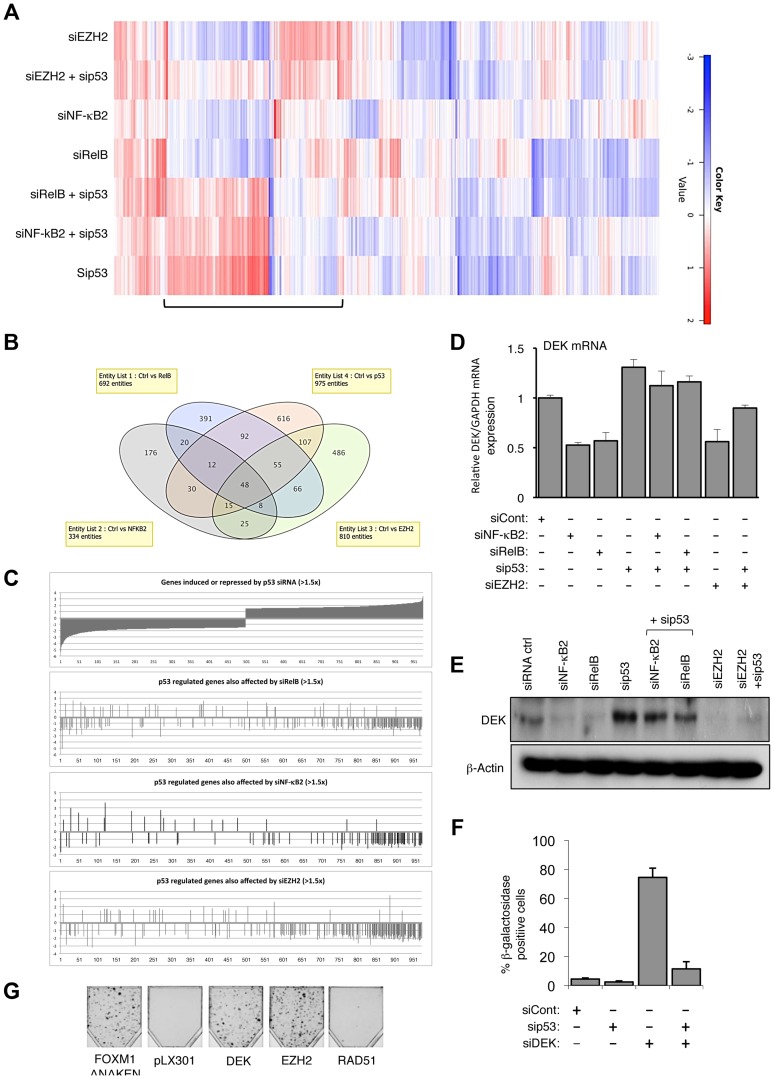
EZH2 is a critical effector of an antagonistic cross-talk between NF-κB and p53. (A) Heatmap showing effects on gene expression of NHD fibroblasts depleted for EZH2, NF-κB2, RelB and p53. NHD fibroblasts were transfected in triplicate with the listed siRNAs. After 48 hours RNA was extracted for microarray analysis. Shown with a bar is the group of genes where EZH2, NF-κB2 and RelB antagonize p53 dependent gene expression. (B) A subset of genes is co-regulated by NF-κB2, RelB, EZH2 and p53 in NHD fibroblasts. (C) Graphical representation of the 975 p53 regulated genes whose expression changes >1.5 fold that are also regulated (>1.5×) by NF-κB2, RelB and Ezh2. (D–E) NF-κB2, RelB, EZH2 regulate DEK expression in NHD fibroblasts. RNA (D) and whole cell protein lysates (E) was prepared from NHD fibroblasts treated with the indicated siRNAs and Q-PCR or western blot analysis of DEK expression was performed. Note that (E) is a reprobing of blots used in Fig. 1C and the β-actin blot shown here is the same as in that figure. (F) siRNA mediated knock down of DEK induces cellular senescence. NHD fibroblasts were transfected with the siRNAs shown and analyzed for senescence after 7 days. (G) EZH2 and DEK alone can rescue senescence. Fibroblasts conditionally immortalized with temperature sensitive T antigen (described in Rovillain et al.) were shifted to the non-permissive temperature and subjected to a clonogenic assay with and without expression of the indicated genes. The panel shows an image of cells upon completion of the assay.

To further investigate the significance of this regulatory pathway and gain insights into the early mechanisms through which these effects were achieved prior to induction of p16^Ink4a^ or p14^ARF^, gene expression profiling was performed on cells 48 hours after transfection with siRNAs targeting NF-κB2, RelB, EZH2 and p53 (accession number for microarray data is E-MTAB-1593). Significantly, a cluster of genes co-regulated by EZH2, NF-κB2 and RelB were also antagonistically regulated by p53, and co-depletion of p53 generally abolished these effects (Fig. 5A&B, see also Figure S5A and Table S1). More detailed analysis of the 975 genes significantly (>1.5 fold) affected by p53 depletion revealed a group of genes normally repressed by p53 (which are therefore induced upon p53 siRNA treatment), antagonistically regulated by EZH2, NF-κB2 and RelB (Fig. 5C, full gene list in Table S2). The potential importance of EZH2 regulation as an ‘effector’ of antagonistic crosstalk between NF-κB and p53 was demonstrated by analysis of the genes where NF-κB and p53 depletions had directly opposing effects: of the 142 RelB/p53 genes in this category, 93 were also regulated by EZH2, while of the 82 genes similarly regulated by NF-κB2/p53, 60 genes were also regulated by EZH2 (Table S2). Interestingly, of the smaller subset of genes regulated in the same manner by NF-κB and p53, that is where both either induce or repress, EZH2 depletion was found to have generally minimal effects (7/54 for RelB/p53 and 1/17 for NF-κB2/p53) (Table S2). The overlap between NF-κB2 and RelB regulated genes was less than expected, given that these factors are often depicted as being favored dimer partners. This most probably results from circumstances where the effect of one dimer partner fell below the 1.5× effect cut off used in this analysis. However, it may also result from compensation by other NF-κB dimer complexes in which these proteins participate, such as p52 homodimer/Bcl3 or p50/RelB complexes.

### Genes regulated by NF-κB/EZH2/p53 are part of a senescence associated gene signature

An NF-κB associated gene signature has been previously described in a study of senescence induced in conditionally immortalized human fibroblasts upon activation of the p16-pRB and p53-p21 tumor suppressor pathways [32]. We were therefore interested in any similarities between the gene signature we had identified and that seen by Rovillain et al., especially as EZH2 expression was also downregulated in this study [32]. Therefore, we integrated our gene expression signature with that of Rovillain et al., to produce a combined heat map (Fig. S5B). This exercise confirmed that a significant proportion of the genes we had previously identified as belonging to the NF-κB2/RelB/EZH2 regulatory network also formed part of the previously identified NF-κB dependent senescence gene signature. Importantly, the heat map reveals that genes co-regulated by EZH2, NF-κB2 and RelB were also antagonistically regulated by p53, and that co-depletion of p53 generally abolished these effects.

To confirm that genes identified as being regulated by the NF-κB/EZH2 pathway did contribute towards suppression of p53 mediated cell senescence, we analyzed the DEK oncogene and histone chaperone that also been described as an inhibitor of senescence [33], [34]. Our microarray analysis revealed its expression to be down-regulated upon NF-κB2, RelB or EZH2 depletion but induced upon treatment with the p53 siRNA, which we confirmed by Q-PCR and western blot analysis (Fig. 5D&E). siRNA depletion of DEK resulted in a striking, p53 dependent induction of senescence (Fig. 5F) but did not affect EZH2 or p53 mRNA levels, consistent with it being a downstream effector of this regulatory pathway (Fig. S5C–E). By contrast, as a control, siRNA depletion of tp53INP1, which is induced upon NF-κB2, RelB or EZH2 depletion but down-regulated upon treatment with the p53 siRNA did not induce senescence or affect senescence induced upon NF-κB2 or RelB depletion (Fig. S5F&G).

The importance of both EZH2 and DEK as regulators of senescence was confirmed by performing a reconstitution experiment, using the conditionally immortalized fibroblast cells described in [32]. Lentiviral gene transfer was used to express DEK and EZH2 in cells at 34°C, before being shifted to 38°C to induce Rb and p53 dependent cellular senescence. A constitutively active FOXM1ΔNΔKEN mutant was included as a positive control [32]. Expression of both EZH2 and DEK proteins was found to suppress p53 and Rb induced senescence, as seen by the formation of colonies in the clonogenic assay (Fig. 5G).

### NF-κB2/RelB/EZH2 regulation of ROS production requires RAC1 and CDC42 activity

**Figure 6:**
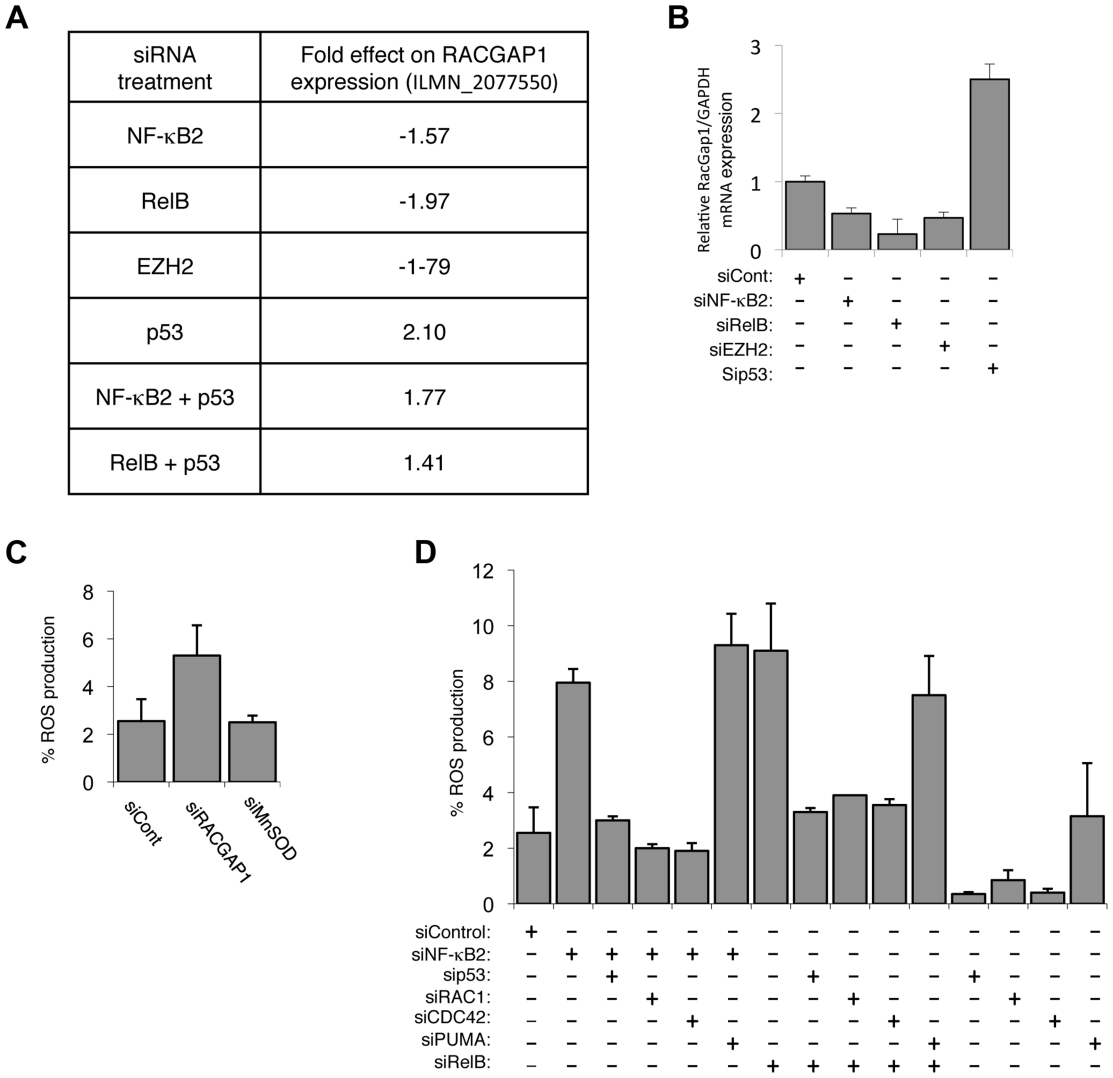
The alternative NF-κB pathway suppresses ROS production through the regulation of RacGAP. (A) Table summarizing the fold effect on RACGAP1 expression induced by transfection of the listed siRNAs in the microarray analysis. (B) NF-κB2, RelB and EZH2 regulate RACGAP1 expression in NHD fibroblasts. RNA was prepared from NHD fibroblasts treated with the indicated siRNAs and Q-PCR analysis of RACGAP1 expression was performed. (C) siRNA mediated knock-down of RACGAP1 induces ROS production. NHD fibroblasts were transfected with the siRNAs shown and analyzed for ROS production after 4 days. (D) ROS production induced by siRNA mediated knock down of NF-κB2 and RelB is dependent upon Rac1 and Cdc42. NHD fibroblasts were transfected with the siRNAs shown and analyzed for ROS production after 4 days.

As described above, p53 dependent generation of ROS is a requirement for senescence induced upon depletion of NF-κB2, RelB and EZH2. We therefore investigated if any of the genes regulated by this pathway could account for these effects. Although NF-κB activity has previously been associated with regulation of ROS levels, we did not find any well-known target genes in our list, such as Manganese Superoxide Dismutase (MnSOD, also known as SOD2). Further analysis using Q-PCR and western blot confirmed that its levels were not significantly changing upon the treatments used in this study (Fig. S6A&B). However, analysis of the genes in Table S2 revealed that expression of Rac GTPase Activating Protein 1 (RACGAP1) fell into the category of genes whose expression was downregulated upon depletion of NF-κB2/RelB/EZH2 and were antagonistically regulated by p53 (Fig. 6A), a result confirmed by Q-PCR (Fig. 6B). Moreover, analysis of the senescence gene expression signature of obtained by Rovillain et al., also showed that RACGAP1 expression was down-regulated upon senescence arrest and reversed upon senescence bypass but was not commented on or further analyzed in that study [32]. RACGAP1 regulates the activity of RAC1 (ras-related C3 botulinum toxin substrate 1) and CDC42 (Cell Division Cycle 42), both members of the RHO family of small GTP binding proteins [35]. Importantly, in the context of this study, both RAC1 and CDC42 can regulate the NADPH oxidase and thereby ROS production [36], [37]. Consistent with the hypothesis that down regulation of RACGAP1 could account, at least in part, for the effects seen on ROS levels, its siRNA depletion resulted in an increase in ROS, while MnSOD did not (Fig. 6C). Moreover, depletion of either RAC1 or CDC42 both inhibited the increases in ROS levels seen upon down regulation of NF-κB2 or RelB, to a similar level seen with the p53 siRNA (Fig. 6D). As a control, no effect was seen with PUMA, a downstream p53 target previously linked to ROS production (Fig. 6D) [12].

Taking these results together with those from Figures 1–5, this demonstrated that regulation of EZH2 by the alternative NF-κB pathway provides a mechanism to control p53 dependent cellular senescence. We next investigated the mechanisms through which regulation of EZH2 by NF-κB is achieved.

### p52 and RelB regulate EZH2 expression through modulation of Rb activity

**Figure 7:**
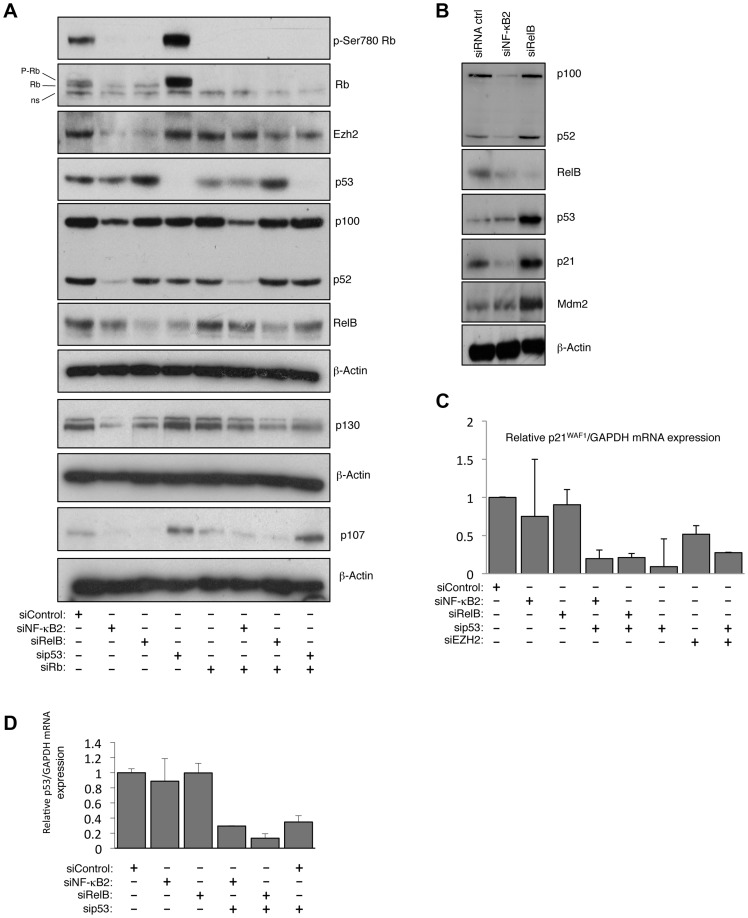
NF-κB2 and RelB regulate EZH2 in an Rb/E2F dependent manner. (A) siRNA mediated knock-down of NF-κB2 and RelB leads to a reduction of Rb- phosphorylation. Western blot analysis of whole cell lysates prepared from NHD fibroblasts 48 hours after transfection with the indicated siRNAs. (B) siRNA mediated knock down of RelB leads to accumulation of p53 and p21^WAF1^ protein level. Western blot analysis of NHD fibroblasts treated with the indicated siRNAs. Whole cell lysates were prepared 48 hours after transfection. (C & D) NF-κB2 and RelB depletion does not affect p21^WAF1^ or p53 mRNA levels. RNA was prepared from NHD fibroblasts treated with the indicated siRNAs and Q-PCR analysis of p21^WAF1^ (C) or p53 (D) expression was performed.

EZH2 expression is regulated by Rb/E2F signaling [19]. We therefore investigated the effect of NF-κB2 and RelB siRNA depletion, and found strong inhibition of Rb phosphorylation, as well as reductions in the levels of Rb family members p107 and p130, (Fig. 7A, Fig. S7A). Moreover, LTβR stimulation also induced Rb phosphorylation (Fig. S7B), consistent with the induction of EZH2 seen before (Fig. 1D & E). Confirming the importance of this pathway, co-depletion of Rb rescued loss of EZH2 expression after treatment with both NF-κB2 and RelB siRNAs (Fig. 7A). Therefore NF-κB2 and RelB regulation of EZH2 is Rb dependent.

### RelB regulates p21^WAF1^ and p53 protein stability

Further investigation revealed unexpected differences between the pathways controlled by RelB and NF-κB2. Interestingly, loss of RelB but not NF-κB2 resulted in a strong induction of p21^WAF1^ and p53 protein (Fig. 7B but see also Fig. 1C). Additional investigation revealed that this was a consequence of an effect of RelB on p21^WAF1^ and p53 protein stability rather than gene transcription: Q-PCR analysis showed that RelB depletion did not significantly affect p21^WAF1^ (CDKN1A) or p53 mRNA levels (Fig. 7C&D, see also Table S3), despite the increase in p53 protein levels. However depletion of p53 in these cells did reduce overall p21^WAF1^ expression (Fig. 7C). This suggested two effects. Firstly that the basal level of p53 in these cells is required for the basal level of p21^WAF1^ expression, while secondly RelB acts to suppress p53 and p21^WAF1^ protein stability, thereby permitting cell proliferation. In this model loss of RelB leads to stabilization of p21^WAF1^ protein, thus inhibiting Rb phosphorylation by Cyclin/CDK complexes, which in turn suppresses E2F induction of EZH2 expression.

### NF-κB2 regulates CDK4 and CDK6 expression

**Figure 8:**
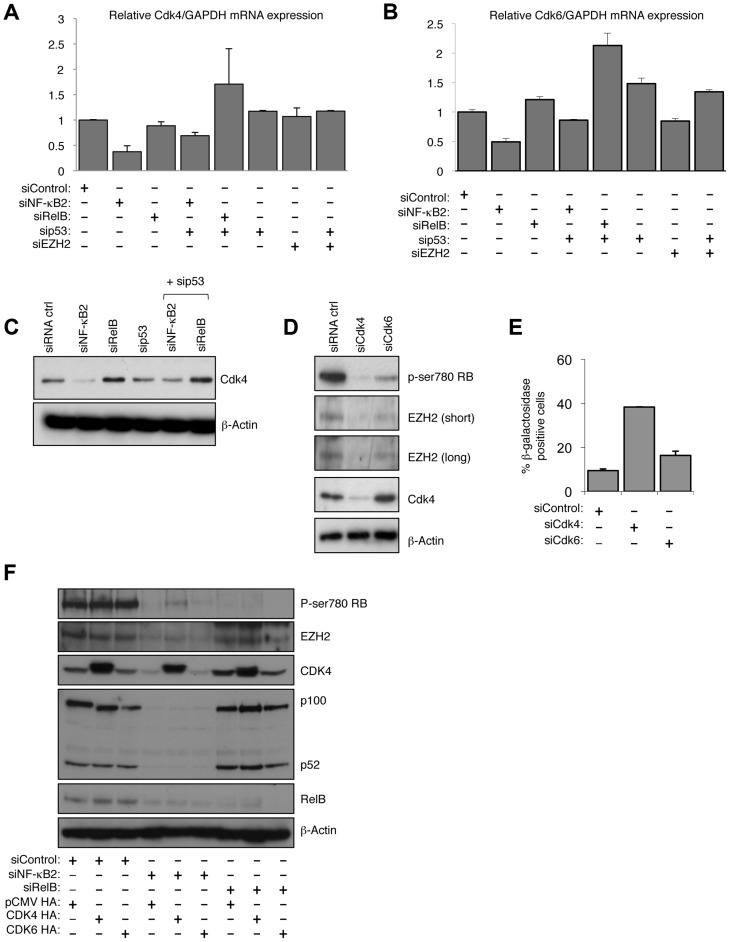
NF-κB2 controls Rb phosphorylation, EZH2 expression and senescence through CDK4 and CDK6 regulation. (A & B) NF-κB2 regulates CDK4 & 6 expression. RNA was prepared from NHD fibroblasts treated with the indicated siRNAs and Q-PCR analysis of CDK4 (A) and CDK6 (B) expression was performed. (C) NF-κB2 regulates CDK4 expression. Western blot analysis of NHD fibroblasts treated with the indicated siRNAs. Note that this is a reprobing of blots used in Fig. 2A and the β-actin blot shown here is the same as in that figure. (D) siRNA mediated knock down of CDK4 and CDK6 results in loss of Rb phosphorylation and EZH2 expression. Western blot analysis of NHD fibroblasts treated with the indicated siRNAs. (E) siRNA mediated knock down of CDK4 induces cellular senescence. NHD fibroblasts were transfected with the listed siRNAs and analyzed for senescence by β-galactosidase staining after 7 days. (F) Re-expression of CDK partially recovers the effects of NF-κB2 siRNA depletion. 96 hours after the transfection of NHD fibroblasts treated with the indicated siRNAs, cells were further transfected with CDK4 and CDK6 expression plasmids. After an additional 24 hours, protein extracts were prepared and western blot analysis performed.

Since depletion of NF-κB2 did not lead to induction of p53 or p21^WAF1^ (Fig. 7B) we investigated if there was an alternative explanation for its effect on Rb phosphorylation. Analysis of our microarray data confirmed that there were no effects on the CDK inhibitors analyzed (Table S3). However, effects on other cell cycle regulatory proteins were seen (Table S4), and in particular we observed NF-κB2 specific downregulation of CDK4 and CDK6, both of which are known to directly phosphorylate Rb [38]. Q-PCR and western blot analysis confirmed that CDK4 and CDK6 expression is selectively lost upon NF-κB2 depletion in NHD fibroblasts, with no effect being seen with the RelB siRNA (Fig. 8A–C). Interestingly, in U2OS cells, we have also observed that CDK4 expression is lost upon siRNA depletion of NF-κB2 [39]. CDK4's key regulatory role as an effector of the effects seen upon depletion of NF-κB2 was confirmed when its siRNA depletion resulted in loss of Rb phosphorylation, reduction in EZH2 expression and induction of senescence (Fig. 8D&E, S8A & B). Furthermore, CDK4 re-expression partially recovered the loss of RB phosphorylation and downregulation of EZH2 expression seen upon depletion of NF-κB2 (Fig. 8F). This was not seen with RelB siRNA treatment.

Chromatin Immunoprecipitation (ChIP) analysis confirmed that both CDK4 and CDK6 are direct p52 target genes in NHD fibroblasts (Fig. S8C&D). Furthermore, demonstrating the generality of this effect, data extracted from a ChIP-Seq analysis of the EBV-transformed human lymphoblastoid B-cell line (LCL) GM12878 [40] confirmed both CDK4 and CDK6 as NF-κB regulated genes (Figs. S8E&F). In GM12878, the EBV-encoded membrane protein LMP1 mimics activated CD40 to stimulate canonical and non-canonical NF-κB pathway activity [40]. Interestingly, this ChIP-Seq analysis revealed that although multiple NF-κB subunits bind the promoters of these genes, including p52, RelB was not found to significantly bind the CDK4 promoter, while p50 was not seen at the CDK6 promoter (Fig. S8E&F). Such differential subunit binding may explain some of the differential effects seen with NF-κB2 and RelB siRNAs.

### RelB regulation of PSMA5 regulates p21^WAF1^ protein stability

**Figure 9:**
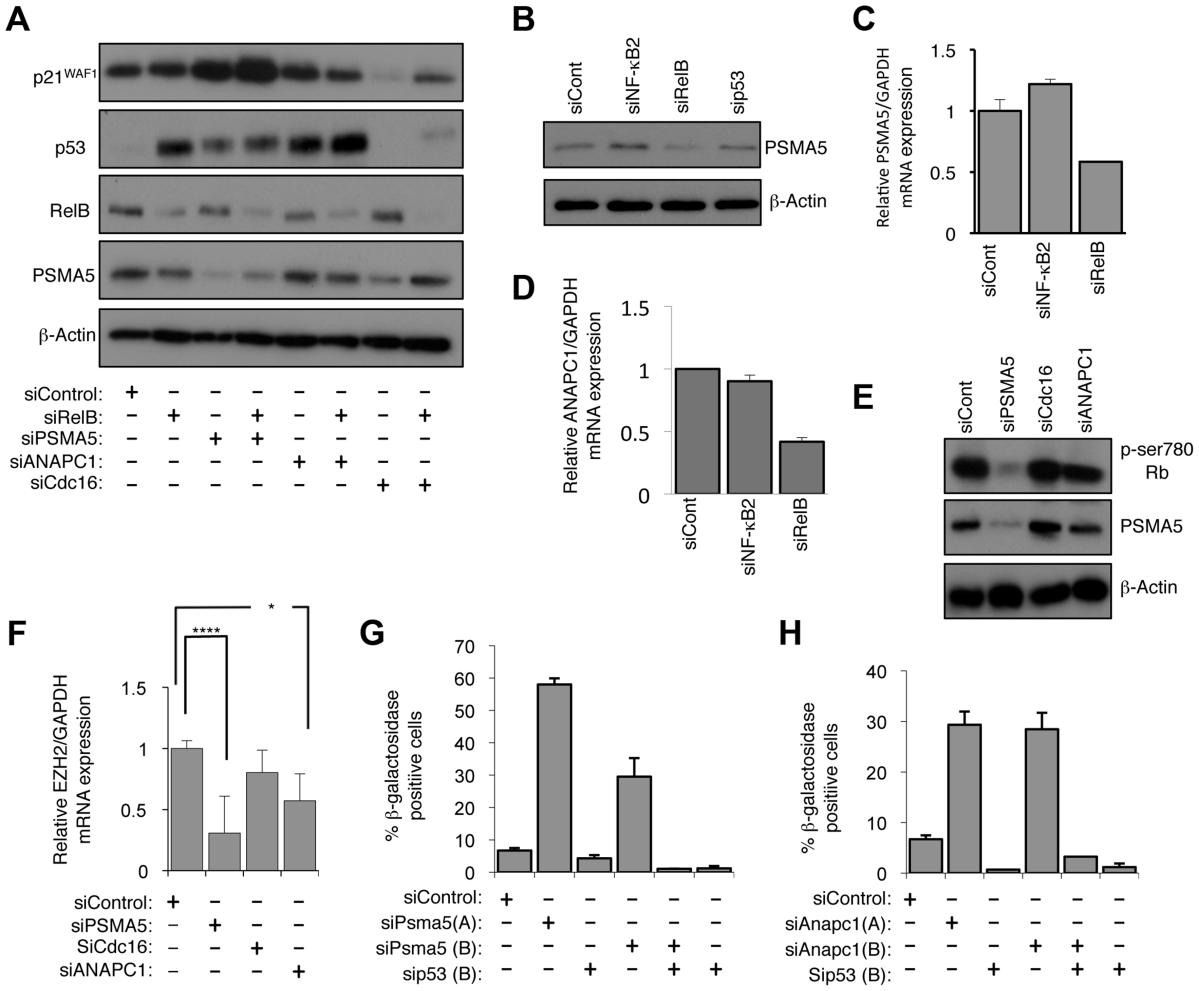
RelB controls Rb phosphorylation, EZH2 expression and senescence through PSMA5 induced regulation of p21^WAF1^ and p53 protein stability. (A) PSMA5 and ANAPC1 regulate p21^WAF1^ and p53 protein stability. Western blot analysis of NHD fibroblasts treated with the indicated siRNAs. (B & C) RelB regulates PSMA5 expression. Whole cell protein lysates (B) or RNA (C) was prepared from NHD fibroblasts treated with the indicated siRNAs and western blot or Q-PCR analysis of PSMA5 expression was performed. (D) RelB regulates ANAPC1 expression. RNA was prepared from NHD fibroblasts treated with the indicated siRNAs and Q-PCR analysis of ANAPC1 was performed. (E) siRNA mediated knock down of PSMA5 results in loss of Rb phosphorylation. Western blot analysis of NHD fibroblasts treated with the indicated siRNAs. (F) siRNA mediated knock down of PSMA5 results in loss of EZH2 expression. RNA was prepared from NHD fibroblasts treated with the indicated siRNAs and Q-PCR analysis of EZH2 was performed. Psma5: (*** p≤0.001) Anapc1: (* p≤0.05). (G & H) siRNA mediated knock down of PSMA5 (G) or ANAPC1 (H) induces p53 dependent cellular senescence. NHD fibroblasts were transfected with the listed siRNAs and analyzed for senescence by β-galactosidase staining after 7 days.

A potential explanation for the effects of RelB on p53 protein stability could result from regulation of Mdm2 levels. Mdm2 is a ubiquitin ligase that induces degradation of p53 and has also been shown to be an NF-κB target gene [41]. However, RelB depletion was seen to consistently induce Mdm2 protein levels (Fig. 1C, 7B), suggesting it is not acting as a RelB effector in this case. We therefore again analyzed our microarray data for RelB regulated genes, whose products are known to have effects on protein stability (Table S5). A number of such genes were identified and a mini-siRNA screen was performed in NHD fibroblasts to ascertain if any had the potential to regulate p53 and p21^WAF1^ stability (Fig. 9A and S9A–C). Particularly striking effects were observed with siRNAs targeting PSMA5 (proteasome (prosome, macropain) subunit, α type, 5) and ANAPC1 (APC1, Anaphase Promoting Complex Subunit 1). Notably, knock down of PSMA5 was the only one to significantly stabilize p21^WAF1^ levels, with the effect of ANAPC1 depletion specifically affecting p53 protein levels (Fig. 9A and S9A). PSMA5 has been previously shown to be transcriptionally upregulated by the antioxidative nuclear Factor E2-related factor 2 (Nrf2) [42] but has not, to the best of our knowledge, been linked to p21^WAF1^ or p53 protein stability, EZH2 or senescence. The Anaphase Promoting Complex (APC/C), of which ANAPC1 is a component, is a cell cycle-regulated E3 ubiquitin ligase. It controls progression through the G1 and M phases of the cell cycle and has been previously associated with senescence induced upon acute loss of the tumour suppressor PTEN [43].

Further analysis confirmed that depletion of RelB but not NF-κB2 resulted in reduced levels of PSMA5 and ANAPC1 (Fig. 9B–D). Interestingly, PSMA5 siRNA depletion resulted in almost complete loss of Rb phosphorylation and a significant reduction in EZH2 mRNA levels (Fig. 9E&F, S9C). By contrast, depletion of ANAPC1 and another control siRNA from our initial screen, CDC16, had no effect on Rb phosphorylation (Fig. 9E) although the former did also affect EZH2 expression (Fig. 9F). This implies that the increase in p53 levels alone seen upon depleting ANAPC1 may also repress EZH2 in an Rb independent manner. Consistent with these effects, siRNA depletion of both PSMA5 and ANAPC1 resulted in significant p53 dependent senescence (Fig. 9G&H).

Similar to our previous results with CDK4/6, ChIP analysis of NHD fibroblasts and analysis of ChIP-Seq data from the EBV-transformed human lymphoblastoid B-cell line (LCL) GM12878 [40] demonstrated that PSMA5 and ANAPC1 are direct NF-κB target genes (Fig. S10A–D).

These results indicate that RelB can regulate numerous genes associated with protein stability. Of these PSMA5 is a key effector of RelB regulation of p21^WAF1^ protein stability, Rb phosphorylation and EZH2 levels, while ANAPC1 contributes to p53 stability and EZH2 repression through an independent pathway.

### EZH2 is a direct NF-κB target gene

**Figure 10:**
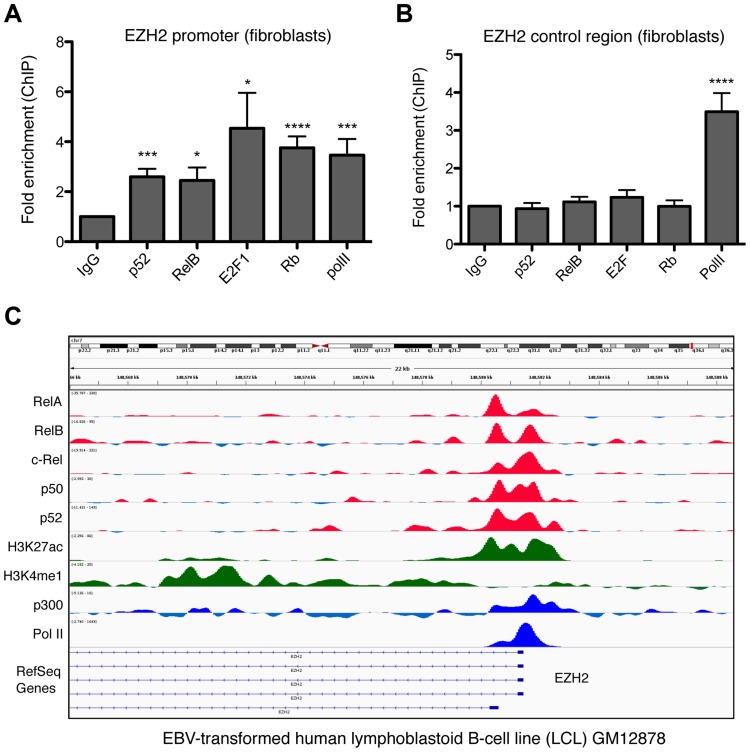
NF-κB subunits bind the EZH2 promoter in fibroblasts and B-cells. (A & B) ChIP analysis of the EZH2 promoter was performed in NHD fibroblast cells using primers close to the core promoter of the EZH2 gene (A) (+596/+888) or an upstream control region (B) (−2802/−2600). Results shown are representative of a minimum of 3 separate experiments. * P≤0.05, ** P≤0.01, *** P≤0.001, **** P≤0.0001. (C) ChIP Seq data showing NF-κB subunit binding in the region of the EZH2 gene in the human EBV-transformed lymphoblastoid B-cell line (LCL) GM12878.

Although these results provided an explanation for the Rb dependent regulation of EZH2 expression by the alternative NF-κB pathway, we also investigated whether EZH2 is also be a direct target for p52 and RelB. ChIP analysis of the EZH2 promoter in NHD fibroblasts confirmed binding by E2F and Rb, as previously reported [19] (Fig. 10A&B). Moreover, this analysis also revealed recruitment of p52 and RelB. This result was confirmed by mining of ChIP-Seq data from GM12878 B-cells [40], where binding of all NF-κB subunits to the EZH2 promoter was seen (Fig. 10C). Taken together these results indicate that p52 and RelB induce EZH2 expression both through regulation of Rb/E2F activity and also directly, through binding the EZH2 promoter.

## Discussion

### NF-κB and senescence

**Figure 11:**
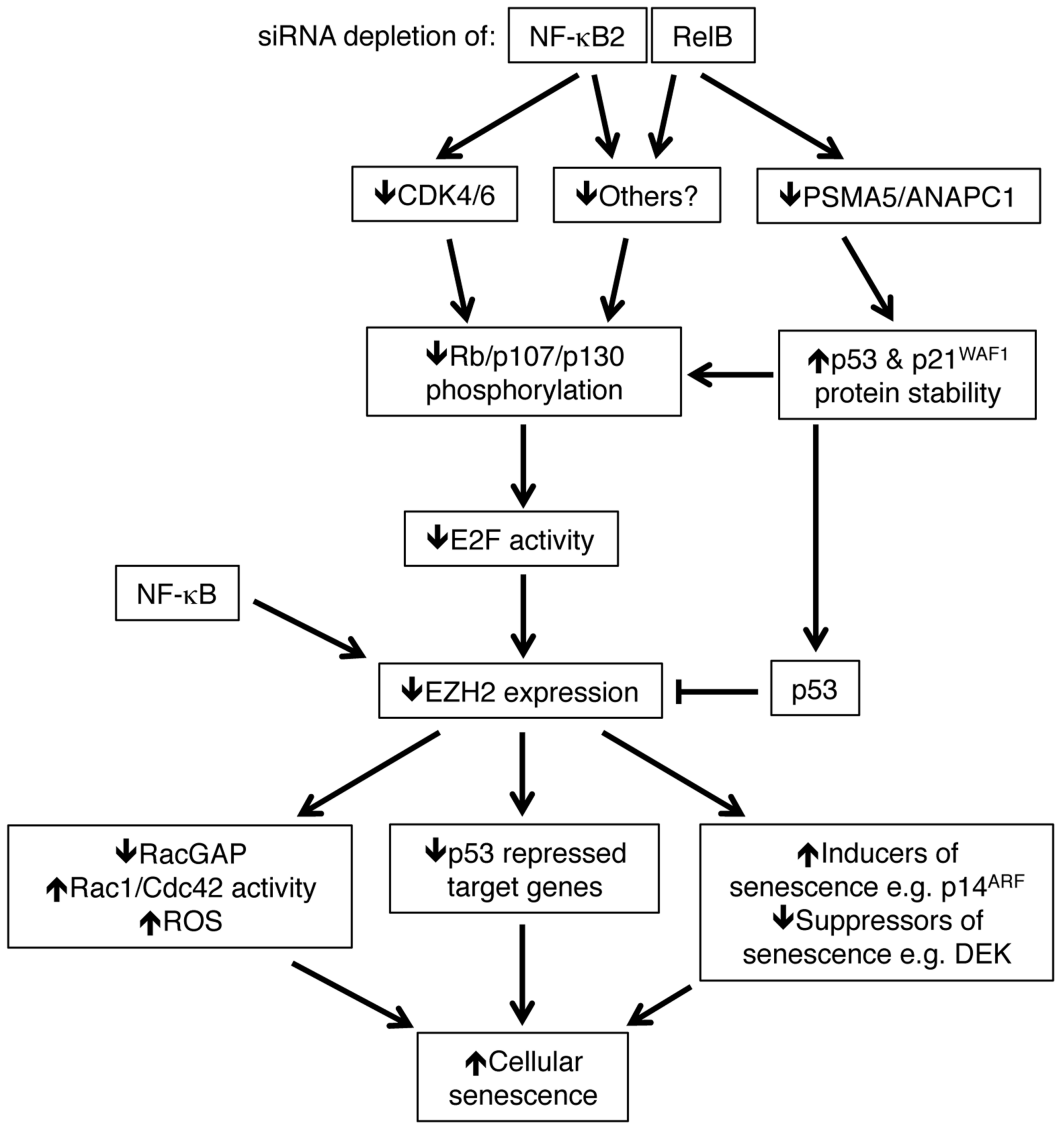
Summary of the results identified in this manuscript through which NF-κB2 and RelB regulate p53 dependent cellular senescence in primary human NHD fibroblasts. Depletion of NF-κB2 and RelB leads to a decrease in Rb phosphorylation. Unphosphorylated Rb represses E2F transcriptional activity and consequently inhibits EZH2 expression (which is negatively regulated by p53). However, this occurs through distinct pathways. Depletion of NF-κB2 leads to down regulation of CDK4 and CDK6, which are known to phosphorylate Rb directly. RelB depletion leads to an increase in p53 and p21 protein stability as a consequence of loss of expression of genes such as PSMA5 and ANAPC1. Other gene targets may be involved in these processes. NF-κB subunits also bind directly to the EZH2 promoter and this may also contribute towards its regulation. As a consequence of this pathway, down regulation of EZH2 results in numerous changes in gene expression, including p53 dependent repression of a number of gene targets. These include inhibition of RACGAP1 expression, resulting in Rac1/Cdc42 dependent induction of reactive oxygen species (ROS). Together with other changes, such as suppression of DEK and ultimately induction of p14^ARF^ and p16^INK4a^, the ultimate consequence of NF-κB2 and RelB siRNA depletion and subsequent loss of EZH2 expression is p53 dependent cell senescence. Note, RACGAP1 activity as well as various inducers/suppressors or senescence may also be regulated by p53. Many of these genes may also be direct targets of NF-κB. Not shown is that oxidative stress is required to drive the basal level p53 activity seen in these cells.

NF-κB activation has previously been associated with induction of senescent cells and the senescence associated secretory phenotype [5]–[7]. However, these studies have focused on the canonical NF-κB pathway and have generally been performed in immortalized or transformed cells. Here we have described a previously unknown pathway through which the alternative NF-κB pathway can suppress cellular senescence. We show that both NF-κB2 and RelB regulate pathways leading to control of Rb phosphorylation and hence determine the level of EZH2 expression (summarized in Fig. 11). Notably, this is achieved through separate but complementary routes, with NF-κB2 regulating expression of CDK4 and CDK6 and RelB regulating the stability of p53 and p21^WAF1^ protein. We demonstrate that this latter effect is achieved through RelB specific regulation of PSMA5 and ANAPC1. It is possible that other NF-κB2 and RelB regulated genes contribute to this process. We then demonstrate that EZH2 antagonizes a subset of p53-regulated genes associated with cell senescence. This included RACGAP1, which through regulating the activity of Rac1 and CDC42, mediated induction of ROS, required for the senescent phenotype. Moreover, through regulating EZH2 expression, this pathway represents the major route of crosstalk between the alternative NF-κB pathway and p53 under these experimental conditions.

Both ChIP analysis of NHD fibroblasts as well as mining of ChIP-Seq data from GM12878 B-cells [40] revealed that CDK4, CDK6, PSMA5 and ANAPC1, together with EZH2 itself, are direct NF-κB target genes. However, it is not clear if this recruitment is a result of direct binding to κB elements or occurs through ‘piggy-backing’ on other transcription factors, such as E2F. The presence of multiple NF-κB subunits on these promoters may also explain why we see differential effects upon knockdown of NF-κB2 and RelB, as there is the potential for other subunits to compensate in ways that may be promoter specific. Moreover, ChIP-Seq analysis showed a lack of recruitment of RelB to the CDK4 promoter (Fig. S8F) in B-cells that may account for why this gene was not seen to be RelB regulated in our experiments. In addition, although p52 and RelB are frequently found in the same NF-κB complex, removal of either subunit will not have the same effect. For example, loss of RelB can still leave p52 homodimers or alternative p52 heterodimers active in the cell. By contrast, RelB does not homodimerise [44] and so loss of p52 can only be potentially compensated for by the activity of p50/RelB heterodimers or other NF-κB complexes.

### Crosstalk between NF-κB and p53

Under normal circumstances, cross regulation between NF-κB and p53, leading to modulation of activity and transcriptional output, also has the potential to influence physiological responses to stress and determine cell fate or behavior under circumstances where both pathways are active. There are a number of situations where such simultaneous activation of both pathways may occur, including many types of DNA damage and oncogene activation. Furthermore, where NF-κB is activated during chronic inflammation, a process shown to promote tumorigenesis, p53 will likely also be induced due to production of ROS [2], [9]–[11]. Indeed we see induction of p53 by both LTβR activation and CD40L stimulation, both inducers of the NF-κB pathway (Fig. 1E, 2B&C, S2B). The consequences of such dual activation may depend on the relative levels of activity of both pathways. In this study, we focused our analysis on the ability of NF-κB to modulate basal level of p53 activity, induced by chronic oxidative stress. Under these conditions, NF-κB activity appears dominant and acts to promote proliferation and suppress senescence. However, under circumstances where p53 is induced to a high level, either by acute administration of a DNA damaging agent or activation of potent oncogene, we propose that the balance would shift and that p53 activity would dominate. Indeed, we have previously observed such an effect where artificial induction of p53 or treatment with ultraviolet light can induce a switch from p52/Bcl-3 complexes to p52/HDAC1 complexes, resulting in a change from activation of Cyclin D1 expression to repression [13]. Both aspects of NF-κB/p53 behavior have physiological relevance and reflect the complex nature of crosstalk between these pathways.

Data in this report also underlines the impact that the activity of tumor suppressors can have on NF-κB dependent gene expression. For example, the effects we see in NHD fibroblasts are dependent upon Rb expression (Fig. 7A) and would not be seen in an Rb null tumor cell line. Similarly, the transcriptional consequences of activation of the alternative NF-κB pathway will differ in cells either with mutant or absent p53. That much research on NF-κB activity in cancer occurs in such cell lines, or is not taken into consideration in model systems, might account for some of the apparently contradictory effects reported in the literature.

### The alternative NF-κB pathway and cancer

The alternative NF-κB pathway can become deregulated in hematological malignancies such as multiple myeloma, through mutation of upstream regulators such as NF-κB inducing kinase (NIK) [45]–[47]. Indeed the NF-κB2 gene itself is subject to translocation, leading to truncation and constitutive processing to p52, in a subset of B and T cell lymphomas [48], [49]. Moreover, the tumor microenvironment can induce alternative NF-κB pathway activity through, for example, the CD40 receptor [45], [46], [50]. Although this branch of NF-κB signalling is associated with the adaptive immune response, the mechanisms through which it can promote tumorigenesis have received little attention and remain poorly defined, compared to the parallel IKKβ/RelA dependent classical pathway. However, due to feed forward mechanisms, in which the classical pathway can prime the activity of the alternative pathway through inducing the expression of the NF-κB2 and RelB genes [4], it is possible that some effects have been assigned to the former pathway but in fact result from the latter.

This pathway provides a mechanism through which deregulated NF-κB2/RelB activity can promote tumorigenesis in cancer cells that retain wild type p53. Consistent with this hypothesis, we show that in CLL, where only 10–15% of tumors at diagnosis contain mutated p53 [51], stimulation of B-CLL cells by CD40L receptor, which induces the alternative NF-κB pathway, results in induction of EZH2 expression (Fig. 2). Moreover, mining of data from ChIP-Seq analysis of the EBV-transformed human lymphoblastoid B-cell line (LCL) GM12878 [40] confirmed that EZH2 is an NF-κB target in this cell type (Fig. 10B). In diffuse large B-cell lymphoma (DLBCL), where activating mutations in EZH2 occur, EZH2 function is essential during B-cell activation and clonal expansion in the germinal center. Recent evidence demonstrated that small molecule inhibition of EZH2 significantly reduced growth of germinal center-derived DLBCL cells, and conditional expression of an EZH2 mutant lymphoma allele was shown to drive lymphomagenesis [52]. These studies highlight that EZH2 has a key role during B-cell activation and together with our data, present the possibility of interplay between NF-κB signaling and EZH2 to enhance survival and proliferation of tumor cells. We propose that this pathway provides a mechanism where activation of NF-κB, either as consequence of the tumor microenvironment or through mutation of upstream signaling pathways (such as occurs in a number of hematological malignancies [53]–[55]), can promote tumorigenesis in cells retaining wild type p53 by suppressing the consequences of p53 activation and providing a window during which further mutagenesis can occur. These effects need not be limited to effects on senescence but may also include suppression of apoptosis, cell cycle arrest and metabolic effects of p53 activity. Our data suggests that inhibition of the alternative pathway, through for example inhibitors of the NIK or IKKα kinases, could have the potential to treat select hematological malignancies that retain wild type p53 and Rb.

## Materials and Methods

### Cells

Primary normal human juvenile dermal fibroblasts were purchased from Promocell (c-12300) and maintained in Fibroblast growth media supplemented with 2% supplement mix (Promocell c-23010) and 1% Pen/Strep/Fungizone Solution (Promocell c-42020). Cells were cultured from passage 2 to passage 10 before being discarded.

CLL cells were cultured with 10 ng/ml IL-4 (R&D Systems, Abingdon, UK). CD40L cell stimulation of CLL cells was performed essentially as described (Pepper et al., 2011). Untransfected L-cells (NTL) and CD40L-expressing mouse fibroblast L-cells were cultured in in RPMI-1640 medium supplemented with 10% foetal bovine serum, 50 units/ml penicillin and 50 mg/ml streptomycin and seeded into 12-well plates (0.6×106/well) and irradiated with 75 Gy. L cells were left to attach for at least 4 h prior to the addition of the CLL cells. The study was approved by the UK NHS Research Ethics Service, and samples were obtained from the Newcastle Haematology Biobank (http://www.ncl.ac.uk/nbb/collections/nhb). Following written informed consent, patients provided peripheral blood samples, from which CLL cells were isolated using Lymphoprep (Axis Shield, Cambridgeshire, UK).

### Inhibitors and treatments

Hydrogen peroxide (H_2_O_2_) was purchased from Sigma (H1009). 3-Deazaneplanocin-A (DZNep) was purchased from Cayman Chemicals (13828). ATM inhibitor was purchased from Tocris (KU55933), Epigallocatechin-Gallate (ECGC) was purchased from Calbiochem (324880), N-acetyl-L-Cysteine (NAC) and IKK-β inhibitor (TPCA-1) were purchased from Sigma (A9165-5gr and T1452).

Concentrations used were: H_2_O_2_ (100 & 200 µM), DZNep: (0.5 µM), KU55933: (10 µM), ECGC: (10 µM), NAC: (5 mM), TPCA-1: (10 µM).

Lymphotoxin β receptor agonist antibody was used at a final concentration used of 2 µG/mL.

### Microarray analysis

Cells were separately transfected in triplicate with siRNAs to generate biological replicates. After 48 hours, RNA was extracted using a PeqLab gold total RNA extraction kit (12-6634-02). Q-PCR and subsequent Principal Components analysis confirmed consistent levels of depletion in all biological replicates, apart from one double EZH2/p53 knockdown, where problems were encountered due to the strong induction of EZH2 levels upon loss of p53 (Table S1). This sample was not included in subsequent analysis. Microarray analysis was performed by Cambridge Genomic Services.

### Bioinformatics analysis

The Illumina Human HT12v4 Expression BeadChip data was background corrected in Illumina Beadstudio, subsequent analysis proceeded using the lumi and limma packages in R (Bioconductor) [56]–[58]. Variant Stabilisation Transform and Robust Spline Normalisation were applied in lumi. Differential expression was detected using linear models and empirical Bayes statistics in limma. A list of genes for each comparison was generated using a Benjamini Hochberg false discovery rate correct p-value of 0.05 and a fold change of 1.5 as cut- offs. Gene lists were integrated with data from Rovillain et al. [32] by comparison across gene names. Genes found in all experiments were retained for inclusion in the integrated heat map.

### Flow cytometric analysis of ROS production

Cells were incubated for 30 minutes with 5 mM 2′,7′ –dichlorofluorescin diacetate (D399 Invitrogen). Cells were then washed twice in phosphate buffered saline (PBS) and resuspended in 200 µl PBS. Samples were analyzed using a FacsCanto flow cytometer (excitation 488 nm, emission 530 nm). Data shown in figures is the average derived from three separate experiments.

### β galactosidase staining

β galactosidase staining was performed according to [59]. Images of senescent stained cells were taken using a Canon power shot A640 camera. The proportion of cells positive for β-galactosidase activity was determined by counting the number of blue cells in the total population, 20 hours after staining. Results shown are averages derived from three separate experiments and error bars indicate the standard deviation.

### CFSE staining

CLL cells were stained with carboxyfluorescein diacetate succinimidyl ester (CFSE) (Life Technologies). These cells were co-cultured, with 10 ng/mL Interleukin 4, on CD40L-expressing fibroblast cells (or on non-CD40L-expressing control (NTL) cells) that had been growth-arrested (with 75 Gy ionising radiation). Quantification of CFSE in CD19+ve cells by flow cytometry [60], was used to show CLL cell proliferation (seen by sub-peaks of CFSE fluorescence).

### Clonogenic assays

Clonogenic assays to measure recovery from senescence were performed essentially as described [32]. Briefly, lentiviral gene transfer was used to express constitutively active FOXM1ΔNΔKEN mutant, DEK, and EZH2 in conditionally immortalized fibroblast cells at 34°C. These cells were then shifted to 38°C, which in control transfected cells induces cellular senescence, causing no colonies to appear in this assay. Colonies were stained with methylene blue.

### Quantitative PCR analysis

Total RNA was extracted with PeqLab gold total RNA extraction kit (12-6634-02), according to the manufacturer's directions. For reverse transcriptase PCR (RT-PCR), 1 µg RNA sample were transcribed with Quantitect Reverse Transcription Kit (QIAgen; 205313). The cDNA stock was diluted by 200 and 5 µl was used for PCR with GoTaq flexi DNA-polymerase (Promega; M8305).

Quantitative PCR data was generated on a Rotor-Gene Q (Qiagen) using the following experimental settings: Hold 50°C for 3 min; Hold 95°C 10 min; Cycling (95°C for 20 sec; 58°C for 20 sec; 72°C for 20 sec with fluorescence measurement)×45; Melting Curve 50–99°C with a heating rate of 1°C every 5 sec. All values were calculated relative to untreated levels and normalized to GAPDH levels using the Pfaffl method [61]. Each RNA sample was assayed in triplicate and the results shown are averages derived from three separate experiments with error bars indicating the standard deviation.

### Luciferase assay

Cells were transfected with siRNAs. 24 hours later, they were transfected with 0.8 µg of pGL3 luciferase reporter vector containing the EZH2 promoter region (Tang et al., 2004) (kind gift of Dr. Tomer Cooks, Weizmann Institute, Israel). After 48 hours, cells were lysed in 100 µl Passive lysis buffer and luciferase was performed using a Dual- Luciferase Reporter Assay System kit (Promega E1910). Luciferase activity was read in a luminometer (Lumat LB9507, Berthold technologies) and normalized to protein content. Results shown are averages derived from five separate experiments and error bars indicate the standard deviation.

### Chromatin Immunoprecipitation (ChIP)

NHD fibroblasts cells, either grown to 70% confluency or analysed 48 hours after transfection, were cross-linked with 1% formaldehyde at room temperature for 10 min. Cells were washed once with cold glycine and then scraped into 0.5 mL of RIPA buffer (0.1% SDS, 1% Triton, 0.5% deoxycholate, 0.5% NP40, 50 mM Tris-HCl pH 7.5, 150 mM NaCl, 50 µg/ml PMSF, 1 µg/ml leupeptin, 1 µg/ml aprotinin, 1 µg/ml pepstatin, Na_3_VO4, 50 µg/ml & 50 µg/ml NaF) and left on ice for 10 minutes. Samples were then sonicated on ice nine times. Each sonication was for 30 seconds with a 30 seconds gap between each sonication. Supernatants were recovered by centrifugation at 12,000 rpm in an eppendorf microfuge for 10 min at 4°C before being diluted 1∶1 in dilution buffer (1% Triton, 2 mM EDTA, 20 mM Tris-HCl pH 8.1, 150 mM NaCl supplemented with 0.1% NP40, protease and phosphatase inhibitors). Samples were then precleared for 2 hours at 4°C with sheared salmon sperm DNA (1 µg/ml) and 20 µl of protein A and G-agarose beads. At this stage, 20 µl of the material was kept as Input material. Immunoprecipitations were performed overnight with specific antibodies (2 µg). The immune complexes were captured by incubation with 20 µl of protein A and G-agarose beads and salmon sperm DNA (1 µg/ml) for 1 hour at 4°C. The immunoprecipitates were washed sequentially for 5 minutes each at 4°C in TSE 1(0.1% SDS, 1% Triton, 2 mM EDTA, 20 mM Tris-HCl pH 8.1,150 mM NaCl), TSE 2 (0.1% SDS, 1% Triton, 2 mM EDTA, 20 mM Tris-HCl pH 8.1,500 mM NaCl), Buffer 3 (250 mM LiCl, 1% NP40, 1% deoxycholate, 1 mM EDTA, 10 mM Tris-HCl pH 8.1 and TE buffer (1 mM EDTA, 10 mM Tris-HCl pH = 8.1). Beads were then eluted with 500 µl of Elution Buffer (1% SDS, 100 mM NaHCO3).

To reverse the crosslinks, samples, including ‘Input’, were incubated at 65°C overnight in a waterbath with 0.2M NaCl. DNA was ethanol precipitated following Phenol-Chloroform extraction. For PCR, 5 µl of DNA was used from an 80 µl DNA preparation and subjected to 40 cycles of PCR amplifications.

For all ChIP results shown are averages derived from three separate experiments and error bars indicate the standard deviation.

### ChIP-Seq

ChIP Seq data shown here was extracted from a previously published analysis of the EBV-transformed lymphoblastoid B-cell line (LCL) GM12878 using validated anti-RelA, RelB, cRel, p52 and p50 antibodies [40]. GM12878 are one of three ENCODE project Tier 1 cell lines. It is an original HapMap cell line used in many genetic studies including the 1000 Genomes Project and has a relatively normal karyotype. Reads from biological replicate ChIP-seq experiments were mapped to the hg19/GRCh37 build of the human genome using bowtie v0.12.8 [62]. ChIP-Seq binding profiles were visualized by the Integrated Genome Viewer (IGV) [63].

### Plasmids

Cdk4-HA (no. 1876), Cdk6-HA (no. 1868) and EZH2-HA (no. 24230) expression plasmids were purchased from Addgene.

### Antibodies

Antibodies used were: anti-EZH2 (3147S Cell Signaling), anti-p52/p100 (05-361 Millipore), anti-RelB (4954S Cell Signaling), anti-p53 (DO-1 sc-126 Santa Cruz), anti-β- Actin (A5441, Sigma), anti-p21 (sc-397 Santa Cruz), anti-Rb (sc-50 Santa Cruz), anti- Cdk4 (sc-260 Santa Cruz), anti-DEK (610948 BD transduction Laboratories), anti-Bcl3 (PA1-41087 Pierce), anti-Lamin B1 (sc-374015 Santa Cruz), anti-p50 (3035S Cell Signaling), anti-MDM2 (OP46 Calbiochem), anti-p130 (610261 BD transduction Laboratories), anti-p107 (sc-318 Santa Cruz), anti-PSMA5 (2457S Cell Signaling), anti-p14ARF (14PO2 Calbiochem), anti-p16INK4a (sc-56330 Santa Cruz), anti MnSOD (sc-133134 Santa Cruz). Phospho-antibodies used were S15-p53 (9284S Cell Signaling) and S780- Rb (8180S Cell Signaling). Lymphotoxin β receptor agonist antibody (anti-HuLTβR:Fc Ab) was a kind gift of Prof. Carl Ware (Sanford/Burnham Medical Research Institute) [64].

### Other procedures

Transfections of siRNAs were performed when cells were at low (<50%) confluency, essentially as described previously [14]. Western blots shown are representative of at least 3 separate experiment and were performed as described [14] using 15–25 µg of protein extracts.

Details of oligonucleotides, siRNAs and primer sequences can be found in Supporting information (Text S1).

### Accession numbers

Microarray data has been submitted to ArrayExpress with accession number is: E-MTAB-1593.

NF-κB ChIP-seq datasets have been published [40] and are deposited in the gene expression omnibus, accession code GSE55105.

## Supporting Information

Figure S1 (A) The basal level p53 protein in NHD fibroblasts is ROS dependent, while constitutive processing of p100 to p52 is ROS independent. NHD fibroblasts were treated with ECGC for 7 days where indicated and Western blot analysis was performed. (B) p53 protein basal and induced level in fibroblast is ATM dependent. NHD fibroblasts were transfected with the siRNAs shown and treated with an ATM inhibitor 48 hours later. 7 days after the initial transfection, whole cell lysates were prepared and western blot analysis was performed. (C) Hydrogen peroxide treatment in NHD fibroblasts induces p53 and reduces the processing of p100 to p52. NHD fibroblasts were treated with the indicated doses of hydrogen peroxide. 7 days after the initial treatment, whole cell lysates were prepared and western blot analysis was performed. (D) Hydrogen peroxide treatment induces cellular senescence in NHD fibroblasts. NHD fibroblasts were treated with the indicated doses of hydrogen peroxide and 7 days after the initial treatment, senescence was measured by β-galactosidase staining. (E) Multiple siRNAs targeting NF-κB2 and RelB result in down regulation of EZH2 levels. Whole cell lysates were prepared 48 hours after siRNA transfection and 20 µg were subjected to SDS- PAGE and western blot analysis. (F) siRNAs targeting NF-κB2 and RelB are specific. RNA was prepared from NHD fibroblasts treated with the indicated siRNAs and Q-PCR analysis of NF-κB2 and RelB expression was performed. (G) siRNA mediated knock-down of Bcl3 leads to a reduction in EZH2 mRNA level. RNA was prepared from NHD fibroblasts treated with the indicated siRNAs and Q-PCR analysis of EZH2 expression was performed. (H) siRNA mediated knock-down of NF-κB2 and RelB leads to a reduction of promoter activity of EZH2. Luciferase assay of NHD fibroblasts treated with the indicated siRNAs and transfected with a pGL3 luciferase reporter vector containing the EZH2 promoter region. Due to the difference in scale, results with p53 and p21^WAF1^ siRNAs are plotted separately. * P≤0.05, ** P≤0.01, *** P≤0.001, **** P≤0.0001.
(TIF)

Figure S2 (A) CD40L stimulation induces CLL cell proliferation. CFSE-labelled CLL cells were either co-cultured on irradiated (75 Gy) CD40L expressing fibroblasts and or control (NTL) cells, both in the presence of IL-4 (10 ng/ml). Each peak, of decreased fluorescence, represents a round of proliferation. No proliferation is observed with co-culture with the NTL cells, remains as the original labelled single peak. CD40L stimulated cells are shown in black, while NTL control cells are shown unfilled. Representative data from day 7 and day 9 after stimulation is shown. (B) Analysis of EZH2 protein level in CLL cells. Western blot analysis of CLL whole cell lysates derived from four different patients (0204, 0205, 0206, 0207) stimulated with CD40L and IL4 where indicated for 24 hours. Cytogenetic analysis confirmed that patient 0205 has del(17p), removing one p53 allele, while the high basal level of p53 in these extracts suggests the other allele is mutant. The identity of the band seen in control cells for patient 0207 is not known and has an apparent molecular weight higher than p53 (the p53 band is indicated with an arrow). Cytogenetically the p53 gene appears normal in these cells. Extracts were prepared using Phosphosafe buffer (Novagen/Millipore).
(TIF)

Figure S3 (A) Multiple siRNAs targeting NF-κB2 and RelB induce cellular senescence. NHD fibroblasts were transfected with the listed siRNAs and analysed for senescence by β- galactosidase staining after 7 days. Blue cells were counted and the percentage of positively staining cells are shown. (B) siRNAs targeting NF-κB2, RelB and Bcl3 result in down regulation of Lamin B1 levels. Western blot analysis of NHD fibroblasts treated with the indicated siRNAs. (C) siRNA targeting NF-κB2 and RelB induce cellular senescence in an ATM dependent manner. NHD fibroblasts were transfected with the listed siRNAs, treated with ATM inhibitor where indicated and analysed for senescence by β-galactosidase staining after 7 days. (D) siRNA mediated knock down of NF-κB2 and RelB induces ROS production. NHD fibroblasts were transfected with the listed siRNAs and treated 48 hours later with NAC. After 1 week they were incubated for 30 minutes with 5 mM DCF-DA and analysed using a FacsCanto. The bar divides cells with high levels of ROS (on the right side), used in data presented in graphical form, from low-level ROS containing cells (on the left side). (E) siRNA mediated knock down of NF-κB2 and RelB induces ROS production. NHD fibroblasts were transfected with the listed siRNAs and treated 48 hours later with ECGC. After 1 week they were incubated for 30 minutes with 5 mM DCF-DA and analysed using a FacsCanto. (F) NF-κB1 depletion does not cause senescence. NHD fibroblasts were transfected with the listed siRNAs. Senescence was measured by β-galactosidase staining after 7 days. Western blot analysis of NHD fibroblasts treated with the indicated siRNAs. (G) siRNA knock down of NF-κB2 and RelB induce ROS production in a p53 dependent manner. NHD fibroblasts were transfected with the siRNAs shown and analyzed for ROS production after 2, 4 and 7 days.
(TIF)

Figure S4 (A) Analysis of senescence in NHD fibroblasts using two different EZH2 siRNAs and a second p53 siRNA. (B) Analysis of p14^ARF^ and p16^Ink4a^ protein levels. Western blot analysis of NHD fibroblasts treated with the indicated siRNAs. (C) p14^ARF^ and p16^INK4a^ RNA level increase after 7days. RNA was prepared from NHD fibroblasts treated with the indicated siRNAs after 48 hours and 7 days. Q-PCR analysis of p14^ARF^ and p16^INK4a^ expression was performed. (D) siRNA knock down of NF-κB2, RelB and Bcl3 induce cellular senescence in an ARF dependent manner.
(TIF)

Figure S5 (A) Principal component analysis of the biological replicates used for microarray analysis. (B) The NF-κB2/RelB/EZH2 regulatory network is part of a senescence gene signature. Gene lists from the microarray analysis in NHDF cells were integrated with data from Rovillain et al. by comparison across gene names. Genes found in all experiments were retained for inclusion in the integrated heat map. (C–E) siRNA mediated knock-down of DEK does not affect EZH2 and p53 mRNA levels. RNA was prepared from NHD fibroblasts treated with the indicated siRNAs and Q- PCR analysis of EZH2 (A), DEK (B) and p53 (C) expression was performed. (F) siRNA mediated knock-down of NF-κB2 and RelB lead to an increase of the RNA level of TP53INP1. RNA was prepared from NHD fibroblasts treated with the indicated siRNAs and Q- PCR analysis of TP53INP1 was performed. (G) siRNA mediated knock-down of TP53INP1 does not affect senescence induced by siRNA NF-κB2 and RelB.
(TIF)

Figure S6 (A–B) NF-κB2, RelB, EZH2 and p53 do not regulate the expression of MnSOD. RNA and whole cell protein lysates were prepared from NHD fibroblasts treated with the indicated siRNAs and Q-PCR (A) or western blot (B) analysis of MnSOD expression was performed.
(TIF)

Figure S7 (A) Multiple siRNAs targeting NF-κB2 and RelB result in down regulation of Rb phosphorylation. Western blot analysis of NHD fibroblasts treated with the indicated siRNAs. Whole cell lysates were prepared 48 hours after transfection and 20 µg were subjected to SDS-PAGE. Please note this is a reprobing of the same blot used in Fig. S1E and so the β-actin control lane is the same. (B) Lymphotoxin β receptor stimulation leads to induction of Rb phosphorylation. NHD fibroblasts were treated with LTβR agonist antibody for the times indicated and western blot analysis was performed to determine Rb phosphorylation. Please note this is a reprobing of the same blot used in Fig. 1E and so the β-actin control lane is the same.
(TIF)

Figure S8 (A & B) Multiple siRNAs targeting CDK4 result in down regulation of EZH2 expression (A) and senescence (B). (C & D) ChIP analysis of p52/RelB binding to the CDK4 and CDK6 promoters was performed in NHD fibroblasts. * P≤0.05, ** P≤0.01, *** P≤0.001, NS - not significant. (E & F) ChIP Seq data showing NF-κB subunit binding in the region of the CDK4 and CDK6 genes in the human EBV-transformed lymphoblastoid B-cell line (LCL) GM12878.
(TIF)

Figure S9 (A) PSMA5 and ANAPC1 regulate p21^WAF1^ and p53 protein stability. Western blot analysis of NHD fibroblasts treated with the indicated siRNAs targeting UBE2C, PSMA5, ANAPC1, CDC16 and FBX5. (B) PSMA5 and ANAPC1 depletion does not affect p21^WAF1^ and p53 mRNA levels. RNA was prepared from NHD fibroblasts treated with the indicated siRNAs and Q-PCR analysis of p21, p53, PSMA5, ANAPC1 and Cdc16 expression was performed. (C) Multiple siRNAs targeting PSMA5 result in upregulation of p21^WAF1^, down regulation of EZH2 expression and loss of Rb phosphorylation. Western blot analysis of NHD fibroblasts treated with the indicated siRNAs. Note data in this figure derives from the same set of protein extracts but resolved on two different gets, with β-actin controls included for both.
(TIF)

Figure S10 (A & B) ChIP analysis of p52/RelB binding to the PSMA5 and ANAPC1 promoters was performed in NHD fibroblasts. * P≤0.05, ** P≤0.01, NS - not significant. (C & D) ChIP Seq data showing NF-κB subunit binding in the region of the CDK4 and CDK6 genes in the human EBV-transformed lymphoblastoid B-cell line (LCL) GM12878.
(TIF)

Table S1 NHD fibroblasts were transfected in triplicates with the listed siRNAs and Q-PCR analysis of NF-κB2, RelB, EZH2, p53 expression was performed. Numbers represent the level of expression upon siRNA treatment compared to the control = 1.
(DOC)

Table S2 Microarray gene expression data showing those genes regulated either in antagonistic or co-operative fashion by NF-κB2, RelB and p53. Values shown are fold effect for control siRNA versus the siRNA indicated. The cut off got this analysis is a 1.5× effect. Where more than one probe set was present on the array, the data shown here is for the one showing the greatest effect. Data for all probe sets can be found in the original microarray data.
(XLSX)

Table S3 Microarray gene expression data for Cyclin D1, Mdm2, and CDK inhibitors.
(DOC)

Table S4 Microarray gene expression data for NF-κB2 regulated genes associated with the cell cycle.
(DOC)

Table S5 Microarray gene expression data for RelB regulated genes associated with ubiquitin mediated degradation.
(DOC)

Text S1 Details of oligonucleotides, siRNAs and PCR primer sequences.
(DOC)
