# Supplementary figures and images for: Regulation of p53 and Rb Links the Alternative NF-κB Pathway to EZH2 Expression and Cell Senescence
Source: PLoS Genet. 2014 Sep 25;10(9):e1004642. doi: 10.1371/journal.pgen.1004642 (PMC4177746; doi:10.1371/journal.pgen.1004642)

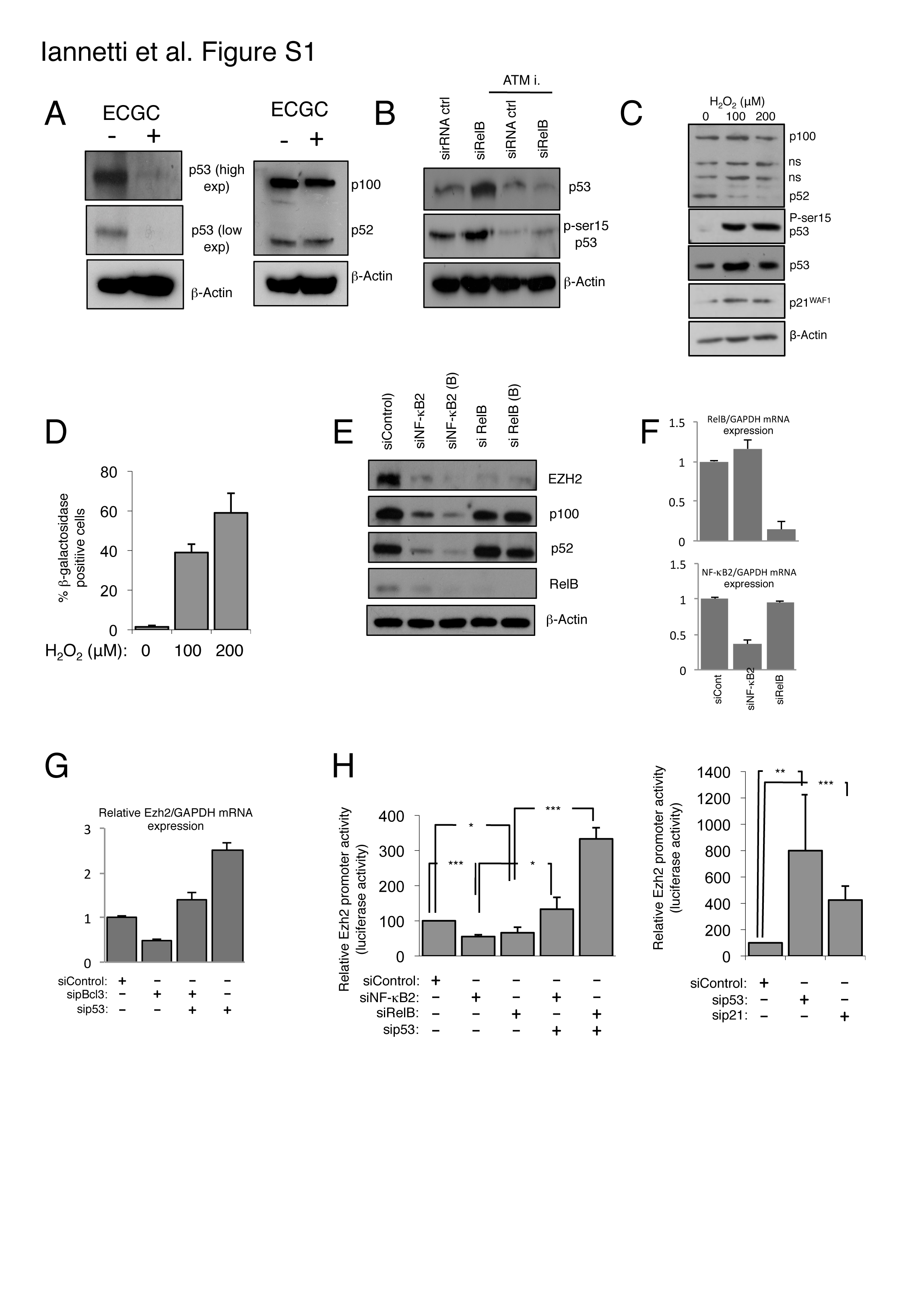

Supplement: Figure S1 — (A) The basal level p53 protein in NHD fibroblasts is ROS dependent, while constitutive processing of p100 to p52 is ROS independent. NHD fibroblasts were treated with ECGC for 7 days where indicated and Western blot analysis was performed. (B) p53 protein basal and induced level in fibroblast is ATM dependent. NHD fibroblasts were transfected with the siRNAs shown and treated with an ATM inhibitor 48 hours later. 7 days after the initial transfection, whole cell lysates were prepared and western blot analysis was performed. (C) Hydrogen peroxide treatment in NHD fibroblasts induces p53 and reduces the processing of p100 to p52. NHD fibroblasts were treated with the indicated doses of hydrogen peroxide. 7 days after the initial treatment, whole cell lysates were prepared and western blot analysis was performed. (D) Hydrogen peroxide treatment induces cellular senescence in NHD fibroblasts. NHD fibroblasts were treated with the indicated doses of hydrogen peroxide and 7 days after the initial treatment, senescence was measured by β-galactosidase staining. (E) Multiple siRNAs targeting NF-κB2 and RelB result in down regulation of EZH2 levels. Whole cell lysates were prepared 48 hours after siRNA transfection and 20 µg were subjected to SDS- PAGE and western blot analysis. (F) siRNAs targeting NF-κB2 and RelB are specific. RNA was prepared from NHD fibroblasts treated with the indicated siRNAs and Q-PCR analysis of NF-κB2 and RelB expression was performed. (G) siRNA mediated knock-down of Bcl3 leads to a reduction in EZH2 mRNA level. RNA was prepared from NHD fibroblasts treated with the indicated siRNAs and Q-PCR analysis of EZH2 expression was performed. (H) siRNA mediated knock-down of NF-κB2 and RelB leads to a reduction of promoter activity of EZH2. Luciferase assay of NHD fibroblasts treated with the indicated siRNAs and transfected with a pGL3 luciferase reporter vector containing the EZH2 promoter region. Due to the difference in scale, results with p53 and p21 [file pgen.1004642.s001.tif]

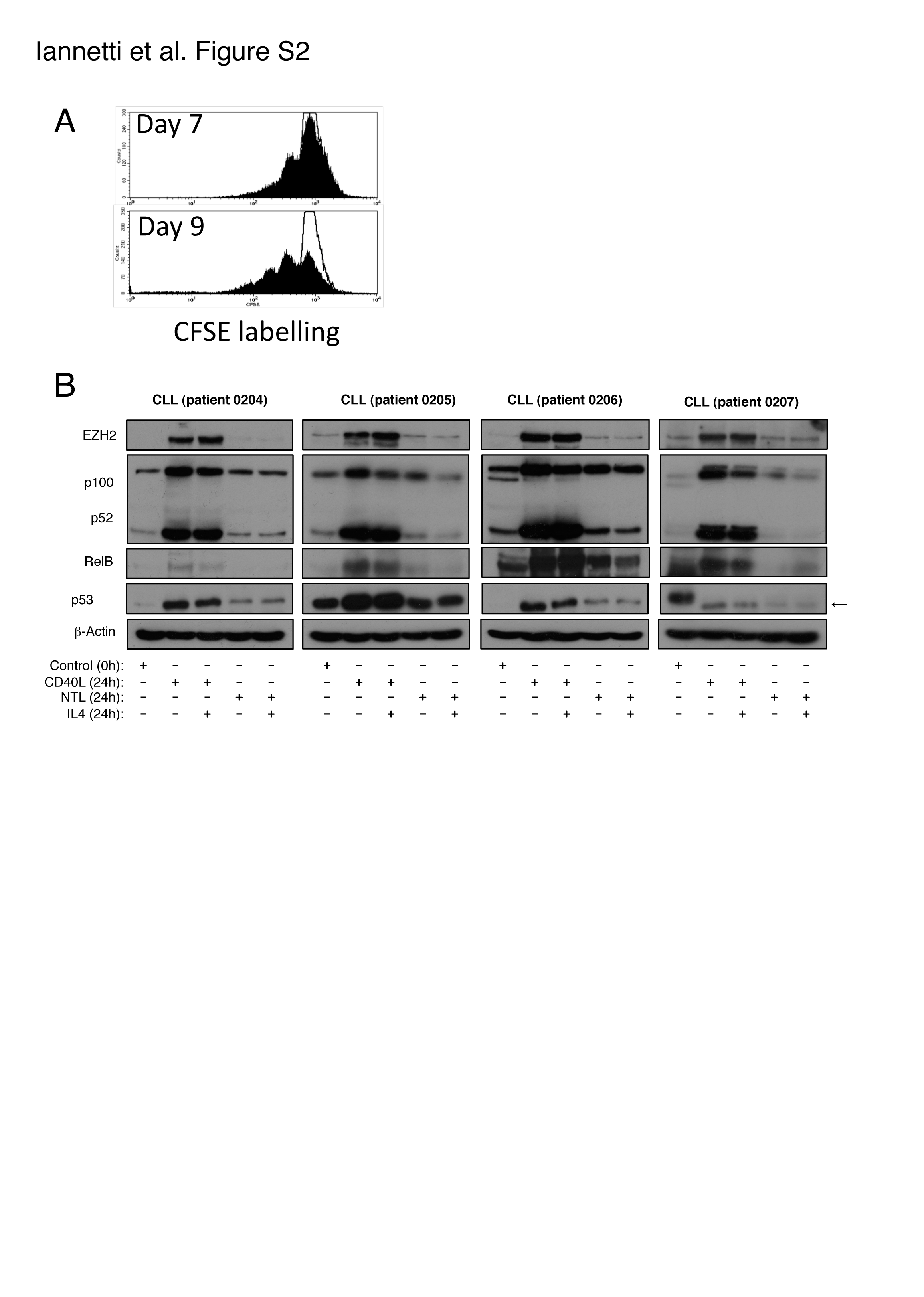

Supplement: Figure S2 — (A) CD40L stimulation induces CLL cell proliferation. CFSE-labelled CLL cells were either co-cultured on irradiated (75 Gy) CD40L expressing fibroblasts and or control (NTL) cells, both in the presence of IL-4 (10 ng/ml). Each peak, of decreased fluorescence, represents a round of proliferation. No proliferation is observed with co-culture with the NTL cells, remains as the original labelled single peak. CD40L stimulated cells are shown in black, while NTL control cells are shown unfilled. Representative data from day 7 and day 9 after stimulation is shown. (B) Analysis of EZH2 protein level in CLL cells. Western blot analysis of CLL whole cell lysates derived from four different patients (0204, 0205, 0206, 0207) stimulated with CD40L and IL4 where indicated for 24 hours. Cytogenetic analysis confirmed that patient 0205 has del(17p), removing one p53 allele, while the high basal level of p53 in these extracts suggests the other allele is mutant. The identity of the band seen in control cells for patient 0207 is not known and has an apparent molecular weight higher than p53 (the p53 band is indicated with an arrow). Cytogenetically the p53 gene appears normal in these cells. Extracts were prepared using Phosphosafe buffer (Novagen/Millipore). (TIF) [file pgen.1004642.s002.tif]

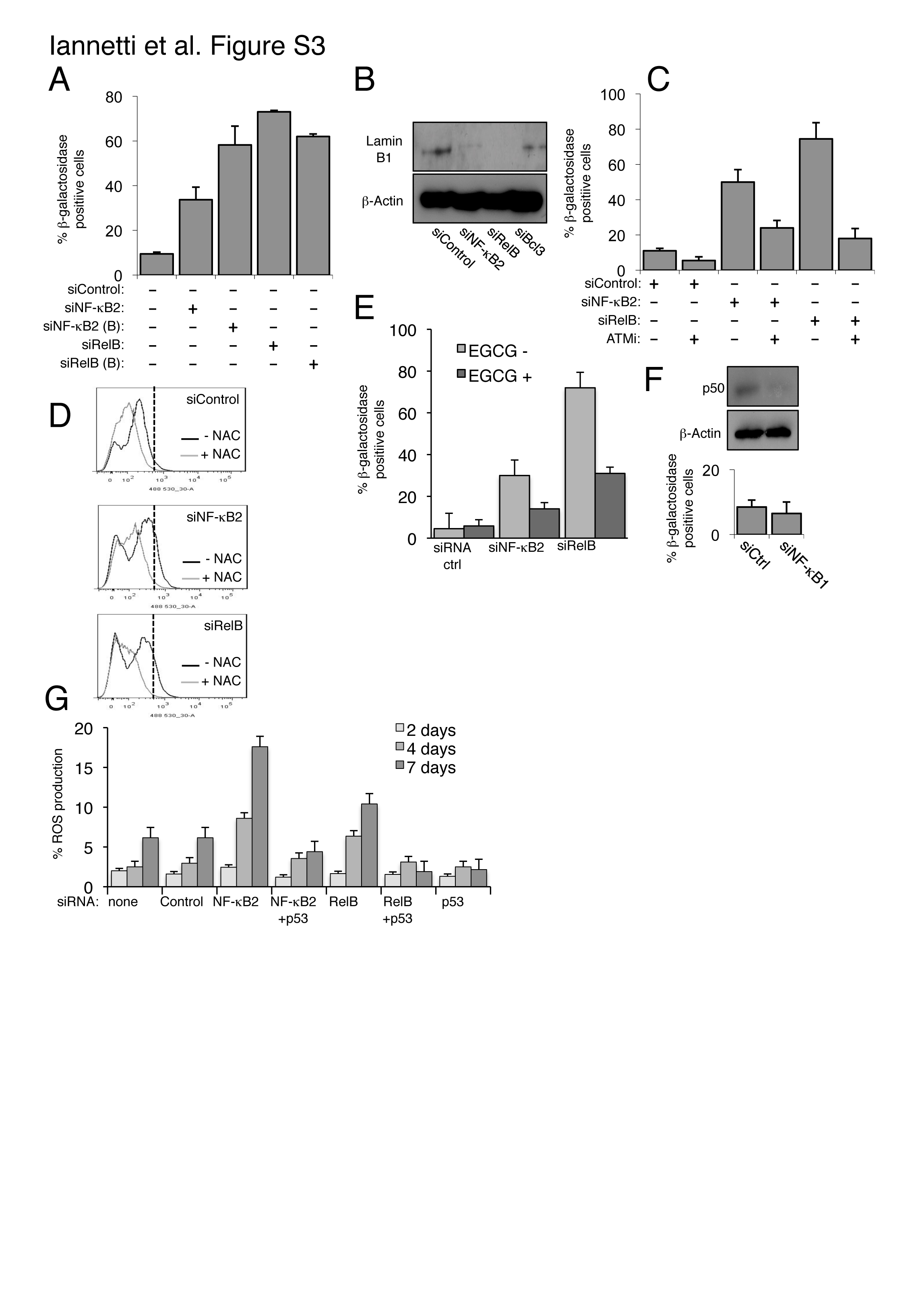

Supplement: Figure S3 — (A) Multiple siRNAs targeting NF-κB2 and RelB induce cellular senescence. NHD fibroblasts were transfected with the listed siRNAs and analysed for senescence by β- galactosidase staining after 7 days. Blue cells were counted and the percentage of positively staining cells are shown. (B) siRNAs targeting NF-κB2, RelB and Bcl3 result in down regulation of Lamin B1 levels. Western blot analysis of NHD fibroblasts treated with the indicated siRNAs. (C) siRNA targeting NF-κB2 and RelB induce cellular senescence in an ATM dependent manner. NHD fibroblasts were transfected with the listed siRNAs, treated with ATM inhibitor where indicated and analysed for senescence by β-galactosidase staining after 7 days. (D) siRNA mediated knock down of NF-κB2 and RelB induces ROS production. NHD fibroblasts were transfected with the listed siRNAs and treated 48 hours later with NAC. After 1 week they were incubated for 30 minutes with 5 mM DCF-DA and analysed using a FacsCanto. The bar divides cells with high levels of ROS (on the right side), used in data presented in graphical form, from low-level ROS containing cells (on the left side). (E) siRNA mediated knock down of NF-κB2 and RelB induces ROS production. NHD fibroblasts were transfected with the listed siRNAs and treated 48 hours later with ECGC. After 1 week they were incubated for 30 minutes with 5 mM DCF-DA and analysed using a FacsCanto. (F) NF-κB1 depletion does not cause senescence. NHD fibroblasts were transfected with the listed siRNAs. Senescence was measured by β-galactosidase staining after 7 days. Western blot analysis of NHD fibroblasts treated with the indicated siRNAs. (G) siRNA knock down of NF-κB2 and RelB induce ROS production in a p53 dependent manner. NHD fibroblasts were transfected with the siRNAs shown and analyzed for ROS production after 2, 4 and 7 days. (TIF) [file pgen.1004642.s003.tif]

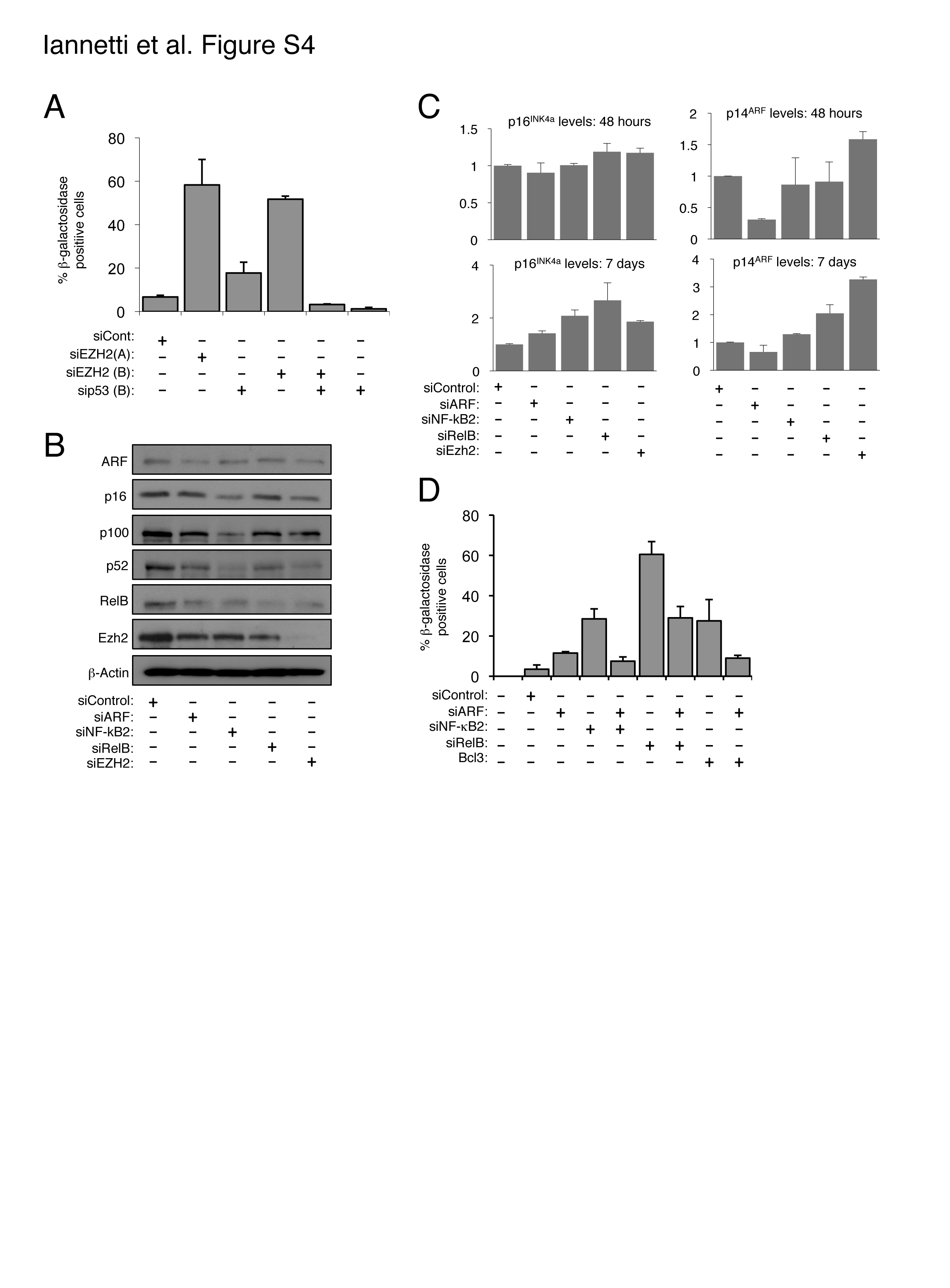

Supplement: Figure S4 — (A) Analysis of senescence in NHD fibroblasts using two different EZH2 siRNAs and a second p53 siRNA. (B) Analysis of p14ARF and p16Ink4a protein levels. Western blot analysis of NHD fibroblasts treated with the indicated siRNAs. (C) p14ARF and p16INK4a RNA level increase after 7days. RNA was prepared from NHD fibroblasts treated with the indicated siRNAs after 48 hours and 7 days. Q-PCR analysis of p14ARF and p16INK4a expression was performed. (D) siRNA knock down of NF-κB2, RelB and Bcl3 induce cellular senescence in an ARF dependent manner. (TIF) [file pgen.1004642.s004.tif]

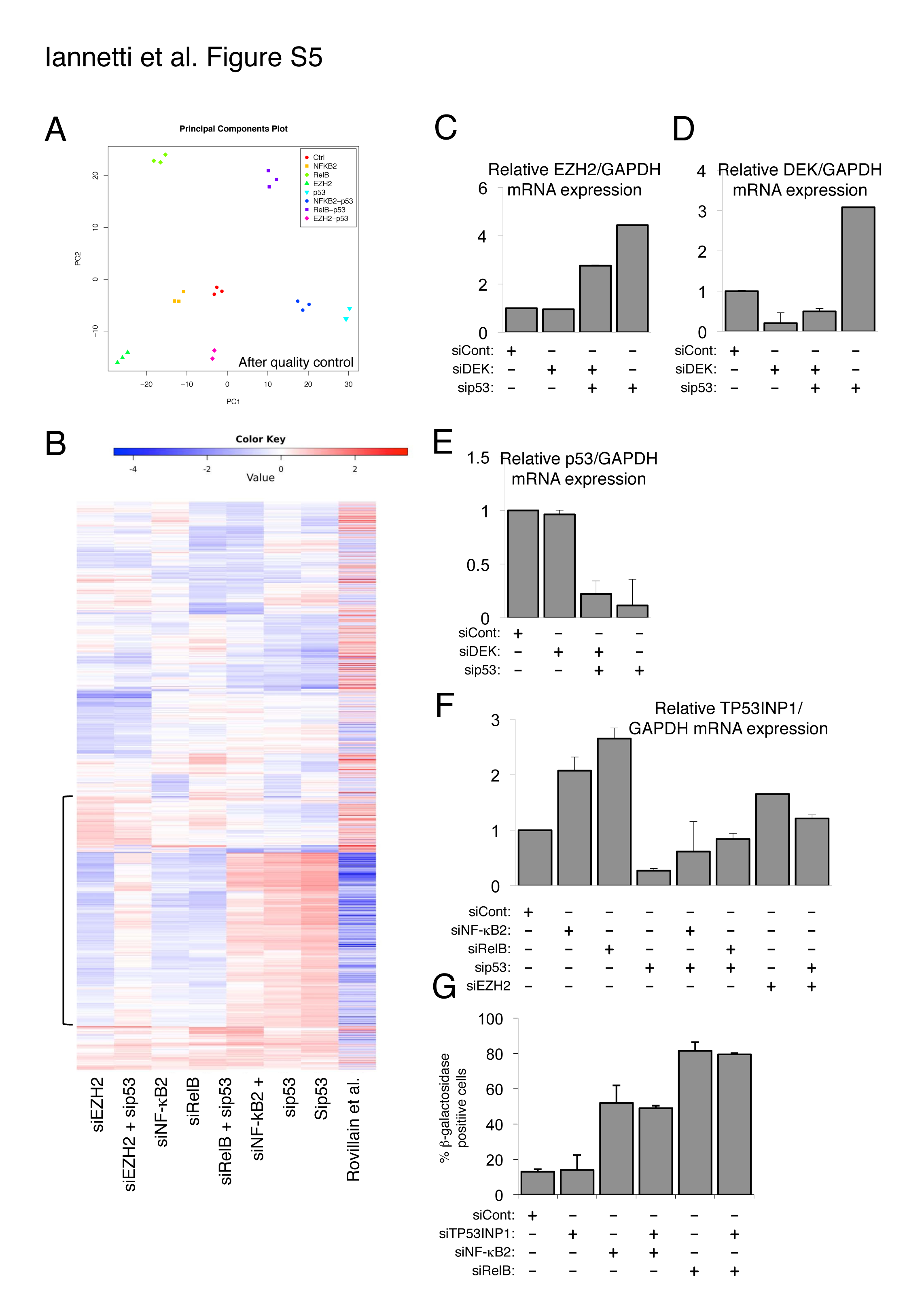

Supplement: Figure S5 — (A) Principal component analysis of the biological replicates used for microarray analysis. (B) The NF-κB2/RelB/EZH2 regulatory network is part of a senescence gene signature. Gene lists from the microarray analysis in NHDF cells were integrated with data from Rovillain et al. by comparison across gene names. Genes found in all experiments were retained for inclusion in the integrated heat map. (C–E) siRNA mediated knock-down of DEK does not affect EZH2 and p53 mRNA levels. RNA was prepared from NHD fibroblasts treated with the indicated siRNAs and Q- PCR analysis of EZH2 (A), DEK (B) and p53 (C) expression was performed. (F) siRNA mediated knock-down of NF-κB2 and RelB lead to an increase of the RNA level of TP53INP1. RNA was prepared from NHD fibroblasts treated with the indicated siRNAs and Q- PCR analysis of TP53INP1 was performed. (G) siRNA mediated knock-down of TP53INP1 does not affect senescence induced by siRNA NF-κB2 and RelB. (TIF) [file pgen.1004642.s005.tif]

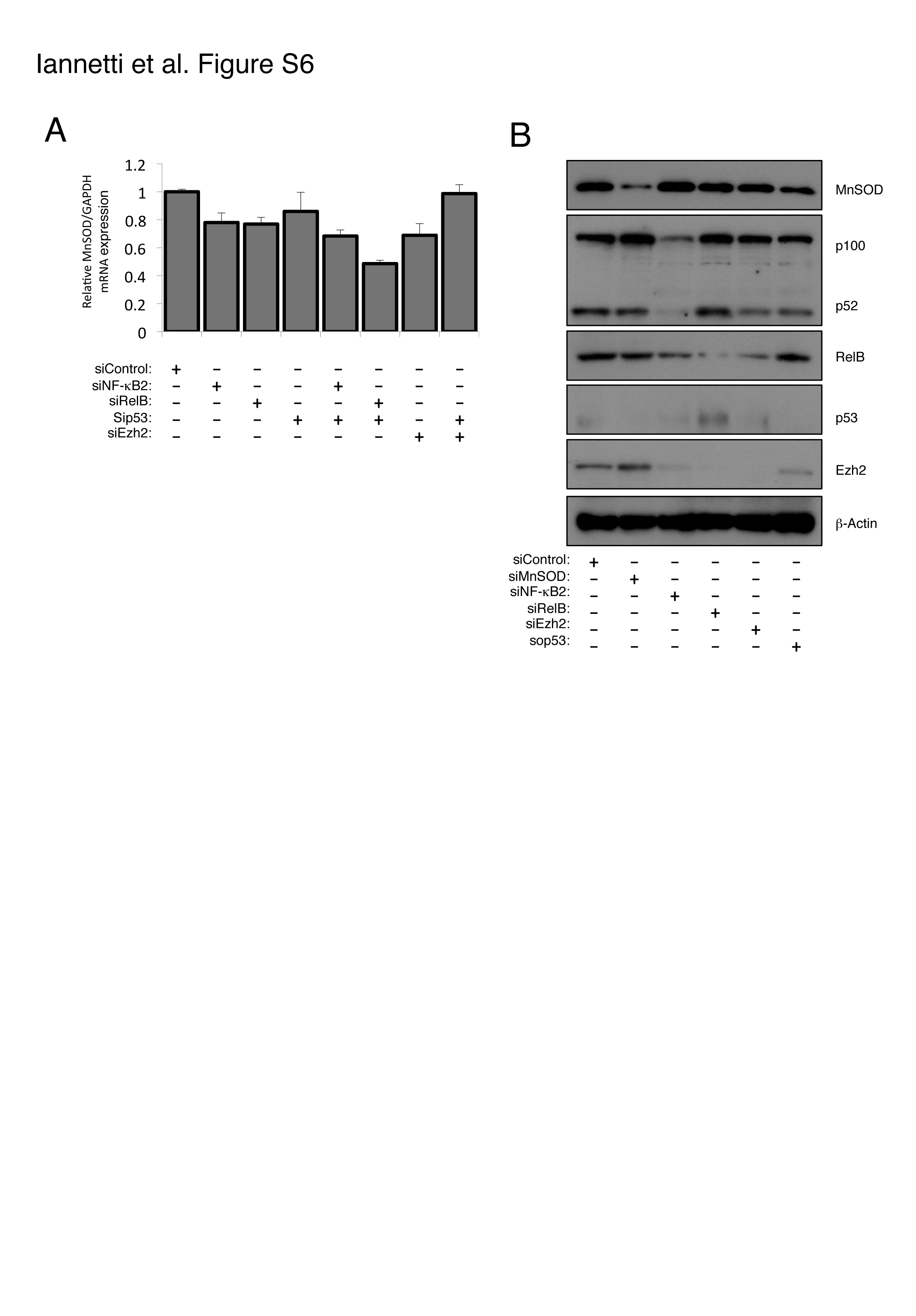

Supplement: Figure S6 — (A–B) NF-κB2, RelB, EZH2 and p53 do not regulate the expression of MnSOD. RNA and whole cell protein lysates were prepared from NHD fibroblasts treated with the indicated siRNAs and Q-PCR (A) or western blot (B) analysis of MnSOD expression was performed. (TIF) [file pgen.1004642.s006.tif]

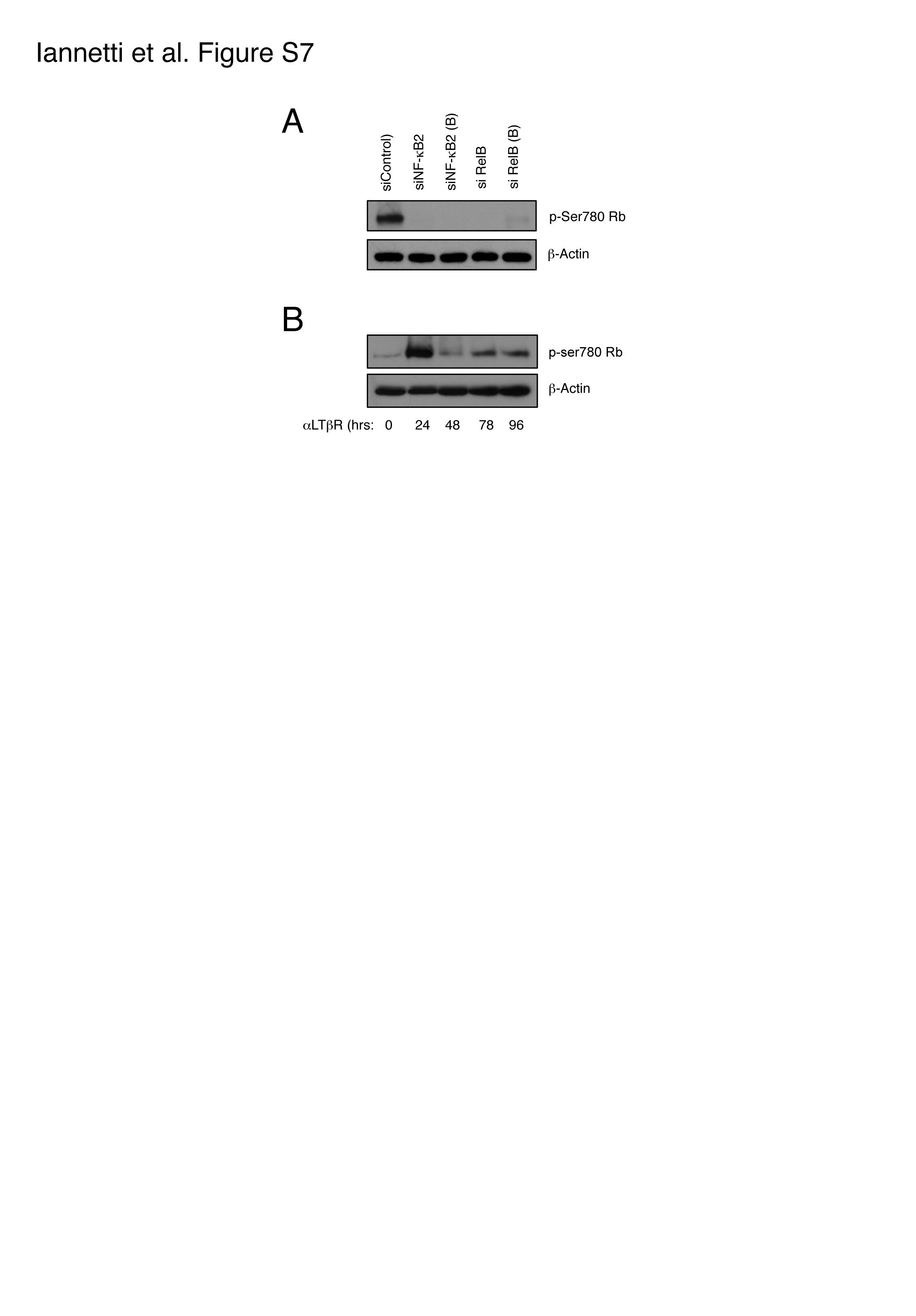

Supplement: Figure S7 — (A) Multiple siRNAs targeting NF-κB2 and RelB result in down regulation of Rb phosphorylation. Western blot analysis of NHD fibroblasts treated with the indicated siRNAs. Whole cell lysates were prepared 48 hours after transfection and 20 µg were subjected to SDS-PAGE. Please note this is a reprobing of the same blot used in Fig. S1E and so the β-actin control lane is the same. (B) Lymphotoxin β receptor stimulation leads to induction of Rb phosphorylation. NHD fibroblasts were treated with LTβR agonist antibody for the times indicated and western blot analysis was performed to determine Rb phosphorylation. Please note this is a reprobing of the same blot used in Fig. 1E and so the β-actin control lane is the same. (TIF) [file pgen.1004642.s007.tif]

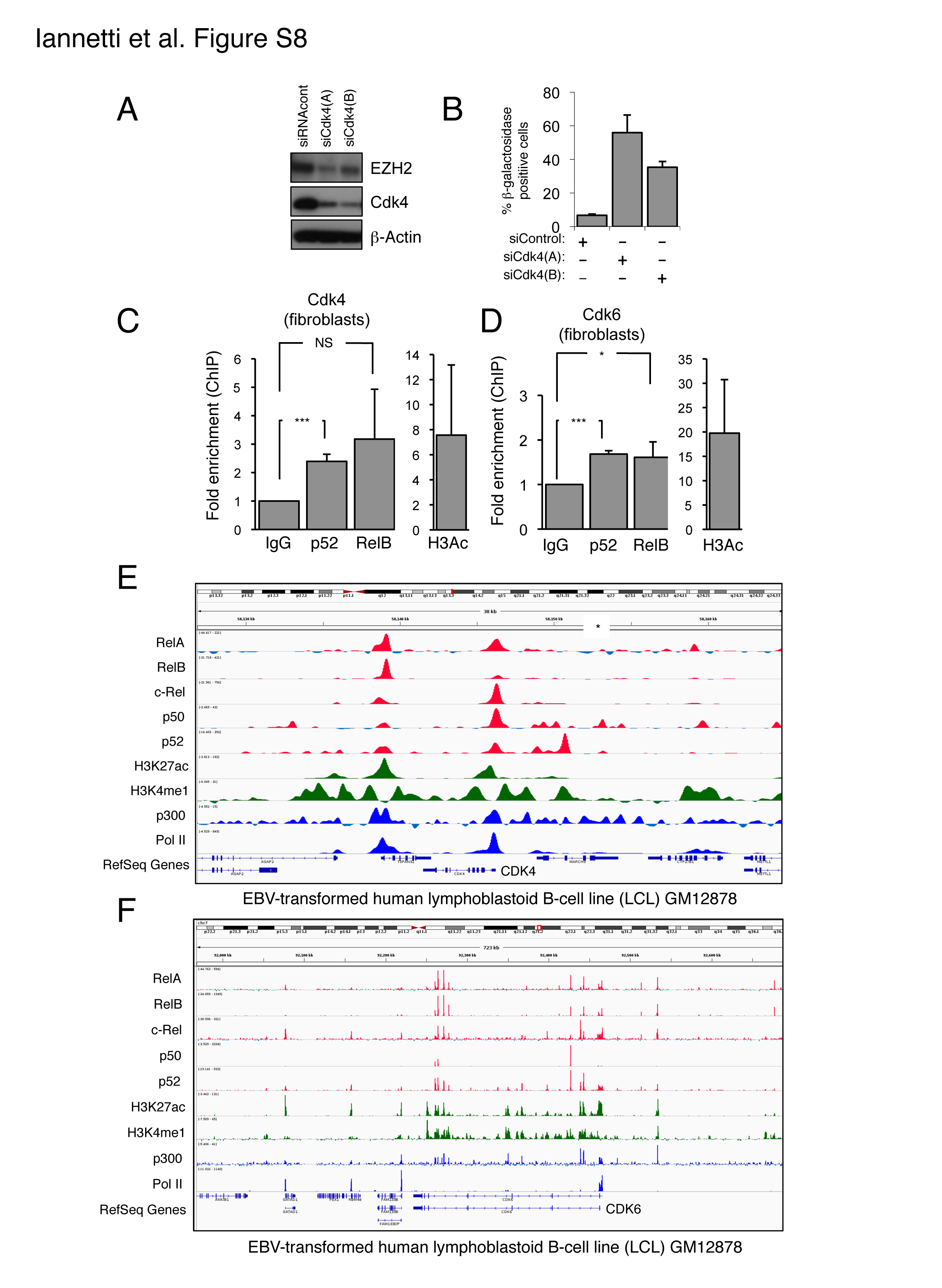

Supplement: Figure S8 — (A & B) Multiple siRNAs targeting CDK4 result in down regulation of EZH2 expression (A) and senescence (B). (C & D) ChIP analysis of p52/RelB binding to the CDK4 and CDK6 promoters was performed in NHD fibroblasts. * P≤0.05, ** P≤0.01, *** P≤0.001, NS - not significant. (E & F) ChIP Seq data showing NF-κB subunit binding in the region of the CDK4 and CDK6 genes in the human EBV-transformed lymphoblastoid B-cell line (LCL) GM12878. (TIF) [file pgen.1004642.s008.tif]

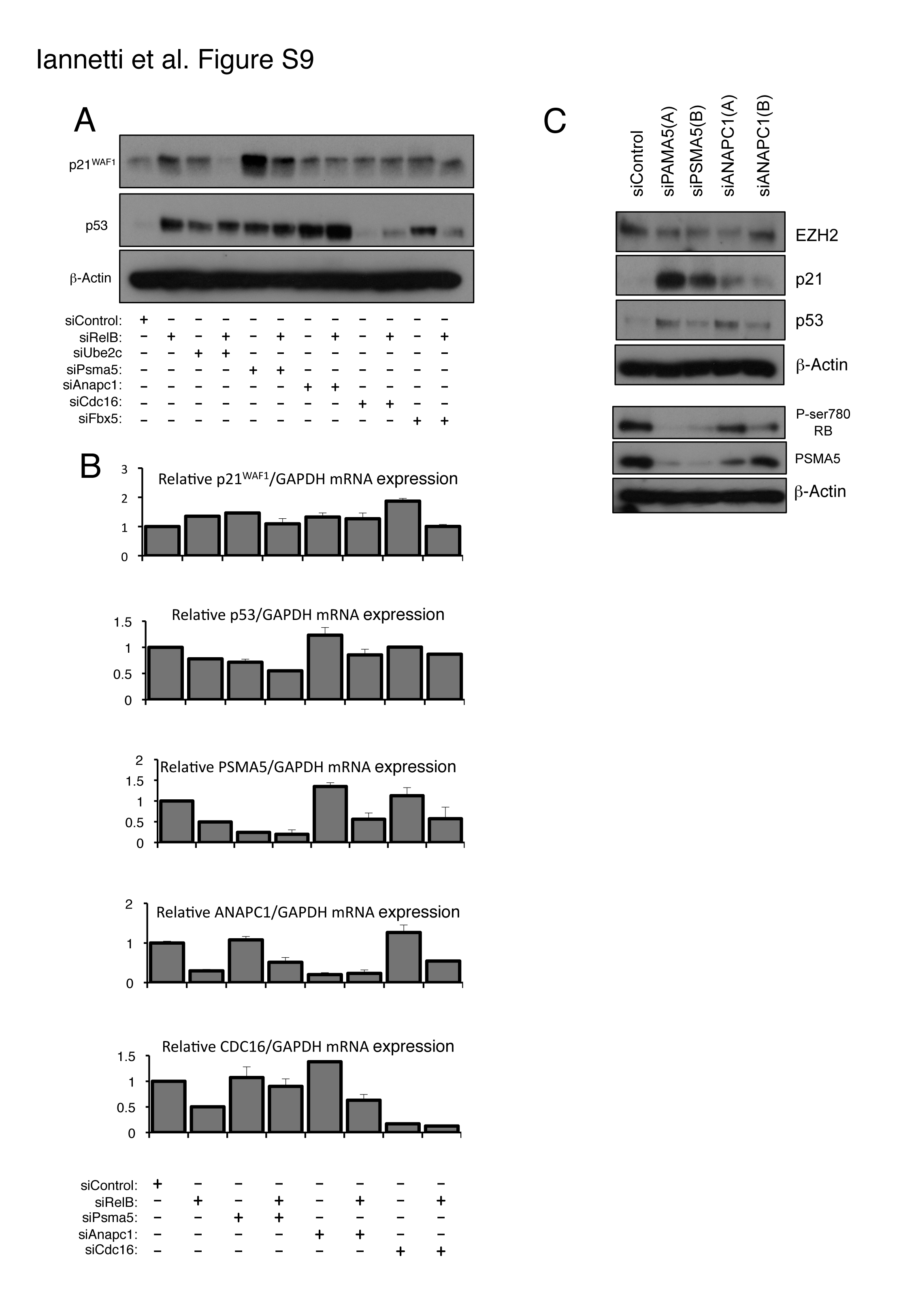

Supplement: Figure S9 — (A) PSMA5 and ANAPC1 regulate p21WAF1 and p53 protein stability. Western blot analysis of NHD fibroblasts treated with the indicated siRNAs targeting UBE2C, PSMA5, ANAPC1, CDC16 and FBX5. (B) PSMA5 and ANAPC1 depletion does not affect p21WAF1 and p53 mRNA levels. RNA was prepared from NHD fibroblasts treated with the indicated siRNAs and Q-PCR analysis of p21, p53, PSMA5, ANAPC1 and Cdc16 expression was performed. (C) Multiple siRNAs targeting PSMA5 result in upregulation of p21WAF1, down regulation of EZH2 expression and loss of Rb phosphorylation. Western blot analysis of NHD fibroblasts treated with the indicated siRNAs. Note data in this figure derives from the same set of protein extracts but resolved on two different gets, with β-actin controls included for both. (TIF) [file pgen.1004642.s009.tif]

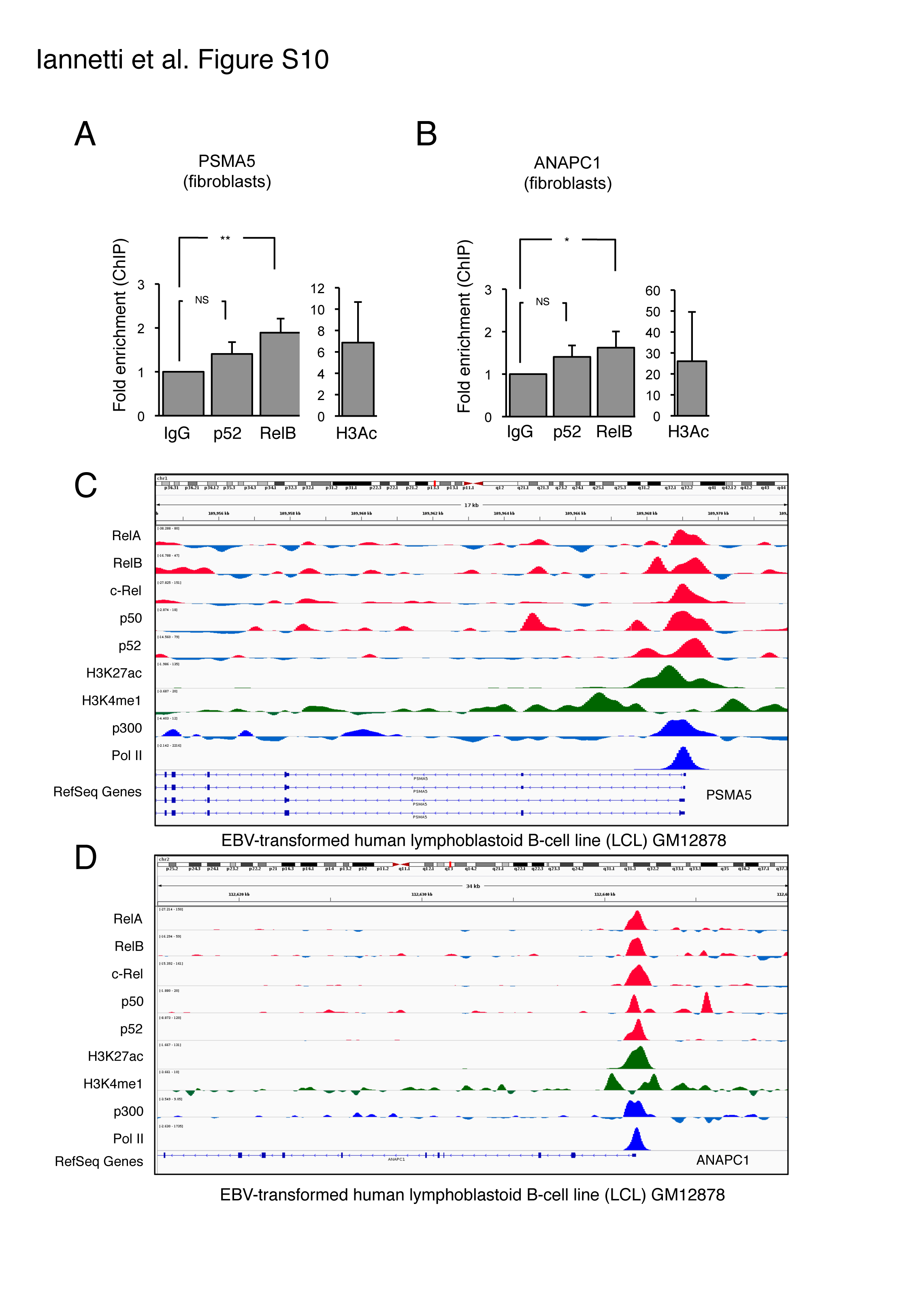

Supplement: Figure S10 — (A & B) ChIP analysis of p52/RelB binding to the PSMA5 and ANAPC1 promoters was performed in NHD fibroblasts. * P≤0.05, ** P≤0.01, NS - not significant. (C & D) ChIP Seq data showing NF-κB subunit binding in the region of the CDK4 and CDK6 genes in the human EBV-transformed lymphoblastoid B-cell line (LCL) GM12878. (TIF) [file pgen.1004642.s010.tif]
