# Supplementary material for: Regulation of p53 and Rb Links the Alternative NF-κB Pathway to EZH2 Expression and Cell Senescence
Source: PLoS Genet. 2014 Sep 25;10(9):e1004642. doi: 10.1371/journal.pgen.1004642 (PMC4177746; doi:10.1371/journal.pgen.1004642)
Supplement: Table S1 — NHD fibroblasts were transfected in triplicates with the listed siRNAs and Q-PCR analysis of NF-κB2, RelB, EZH2, p53 expression was performed. Numbers represent the level of expression upon siRNA treatment compared to the control = 1. (DOC) [file pgen.1004642.s011.doc]

Iannetti et al. Table S1

| **siControl=1** | **siNF-κB2**  **A** | **siNF-κB2**  **B** | **siNF-κB2**  **C** | **siNF-κB2/sip53**  **A** | **siNF-κB2/sip53**  **B** | **siNF-κB2/sip53**  **C** |
| --- | --- | --- | --- | --- | --- | --- |
| Relative NF-κB2/GAPDH mRNA Standard deviation | 0.25  0.02 | 0.23  0.08 | 0.26  0.16 | 0.25  0.01 | 0.28  0.12 | 0.29  0.03 |
| **siControl=1** | **siNF-κB2**  **A** | **siNF-κB2**  **B** | **siNF-κB2**  **C** | **siNF-κB2/sip53**  **A** | **siNF-κB2/sip53**  **B** | **siNF-κB2/sip53**  **C** |
| Relative NF-κB2/GAPDH mRNA Standard deviation | 0.25  0.02 | 0.23  0.08 | 0.26  0.16 | 0.25  0.01 | 0.28  0.12 | 0.29  0.03 |
| **siControl=1** | **siNF-κB2**  **A** | **siNF-κB2**  **B** | **siNF-κB2**  **C** | **siNF-κB2/sip53**  **A** | **siNF-κB2/sip53**  **B** | **siNF-κB2/sip53**  **C** |
| Relative NF-κB2/GAPDH mRNA Standard deviation | 0.25  0.02 | 0.23  0.08 | 0.26  0.16 | 0.25  0.01 | 0.28  0.12 | 0.29  0.03 |
| **siControl=1** | **siNF-κB2**  **A** | **siNF-κB2**  **B** | **siNF-κB2**  **C** | **siNF-κB2/sip53**  **A** | **siNF-κB2/sip53**  **B** | **siNF-κB2/sip53**  **C** |
| Relative NF-κB2/GAPDH mRNA Standard deviation | 0.25  0.02 | 0.23  0.08 | 0.26  0.16 | 0.25  0.01 | 0.28  0.12 | 0.29  0.03 |
| **siControl=1** | **siNF-κB2**  **A** | **siNF-κB2**  **B** | **siNF-κB2**  **C** | **siNF-κB2/sip53**  **A** | **siNF-κB2/sip53**  **B** | **siNF-κB2/sip53**  **C** |
| Relative NF-κB2/GAPDH mRNA Standard deviation | 0.25  0.02 | 0.23  0.08 | 0.26  0.16 | 0.25  0.01 | 0.28  0.12 | 0.29  0.03 |
| **siControl=1** | **sip53**  **A** | **sip53**  **B** | **sip53**  **C** | **siEzh2/sip53**  **A** | **siEzh2/sip53**  **B** | **siEzh2/sip53**  **C** |
| Relative Ezh2/GAPDH mRNA  Standard deviation | 1.91  0.16 | 3.30  0.07 | 2.65  0.25 | 1.31  0.20 | 0.77  0.25 | 0.65  0.01 |
