# Supplementary material for: Regulation of p53 and Rb Links the Alternative NF-κB Pathway to EZH2 Expression and Cell Senescence
Source: PLoS Genet. 2014 Sep 25;10(9):e1004642. doi: 10.1371/journal.pgen.1004642 (PMC4177746; doi:10.1371/journal.pgen.1004642)
Supplement: Table S3 — Microarray gene expression data for Cyclin D1, Mdm2, and CDK inhibitors. (DOC) [file pgen.1004642.s013.doc]

Iannetti et al. Table S3

Microarray gene expression data for Cyclin D1, Mdm2 and CDK inhibitors

| Gene Symbol | Gene Name | siNF-κB2 | siRelB | siEZH2 | sip53 |
| --- | --- | --- | --- | --- | --- |
| CCND1 | cyclin D1 | NR | -1.33 | -2.07 | -1.25 |
| MDM2 | Mdm2, p53 E3 ubiquitin protein ligase homolog (mouse) | NR | 1.18 | NR | -1.23 |
| CDKN1A | cyclin-dependent kinase inhibitor 1A (p21, Cip1) | NR | -1.15 | -1.18 | -1.41 |
| CDKN1A | cyclin-dependent kinase inhibitor 1A (p21, Cip1) | NR | NR | NR | -4.56 |
| CDKN2A | cyclin-dependent kinase inhibitor 2A (melanoma, p16, inhibits CDK4) | -1.17 | NR | NR | NR |
| CDKN2B | cyclin-dependent kinase inhibitor 2B (p15, inhibits CDK4) | NR | 1.24 | 1.22 | NR |
| CDKN2B | cyclin-dependent kinase inhibitor 2B (p15, inhibits CDK4) | NR | 1.30 | 1.42 | NR |
| CDKN1B | cyclin-dependent kinase inhibitor 1B (p27, Kip1) | NR | 1.40 | NR | 1.47 |
| CDKN1B | cyclin-dependent kinase inhibitor 1B (p27, Kip1) | NR | 1.39 | NR | 1.64 |
| CDKN2D | cyclin-dependent kinase inhibitor 2D (p19, inhibits CDK4) | NR | -1.17 | NR | NR |
| CDKN2D | cyclin-dependent kinase inhibitor 2D (p19, inhibits CDK4) | NR | -1.50 | -1.21 | 1.64 |
| CDKN2C | cyclin-dependent kinase inhibitor 2C (p18, inhibits CDK4) | NR | NR | NR | -1.19 |
| CDKN2C | cyclin-dependent kinase inhibitor 2C (p18, inhibits CDK4) | NR | NR | NR | 1.23 |
| CDKN3 | cyclin-dependent kinase inhibitor 3 | -1.44 | -1.70 | -2.06 | 2.07 |

NR = No result
