# Supplementary material for: Regulation of p53 and Rb Links the Alternative NF-κB Pathway to EZH2 Expression and Cell Senescence
Source: PLoS Genet. 2014 Sep 25;10(9):e1004642. doi: 10.1371/journal.pgen.1004642 (PMC4177746; doi:10.1371/journal.pgen.1004642)
Supplement: Table S4 — Microarray gene expression data for NF-κB2 regulated genes associated with the cell cycle. (DOC) [file pgen.1004642.s014.doc]

Iannetti et al. Table S4. NF-κB2 regulated genes associated with the cell cycle

| Gene | Description | siNF-κB2 | siRelB | siEZH2 | sip53 |
| --- | --- | --- | --- | --- | --- |
| MCM3 | minichromosome maintenance complex component 3 | -2.08 | -1.75 | -1.65 | 2.18 |
| CDK7 | cyclin-dependent kinase 7 | -2.03 | NR | NR | 1.19 |
| CDC20 | cell division cycle 20 homolog (S. cerevisiae) | -1.87 | -1.80 | -2.67 | 1.79 |
| CDK6 | cyclin-dependent kinase 6 | -1.84 | 1.29 | 1.29 | NR |
| CCNA2 | cyclin A2 | -1.83 | -2.01 | -2.22 | 2.44 |
| CDK4 | cyclin-dependent kinase 4 | -1.75 | NR | NR | 1.14 |
| CDC45L | cell division cycle 45 homolog (S. cerevisiae) | -1.744 | -1.91 | -2.06 | 1.99 |
| RFC4 | replication factor C (activator 1) 4, 37kDa | -1.74 | -1.77 | -1.90 | 1.70 |
| RAD51AP1 | RAD51 associated protein 1 | -1.73 | -1.87 | -2.10 | 1.99 |
| CDCA5 | cell division cycle associated 5 | -1.70 | -1.89 | -2.03 | 2.31 |
| MCM4 | minichromosome maintenance complex component 4 | -1.68 | -1.36 | -1.69 | 2.17 |
| MCM7 | minichromosome maintenance complex component 7 | -1.66 | -1.52 | -1.83 | 1.99 |
| CDCA3 | cell division cycle associated 3 | -1.65 | -1.49 | -2.14 | 2.26 |
| MCM10 | minichromosome maintenance complex component 10 | -1.63 | -1.59 | -1.640 | 1.48 |
| MCM2 | minichromosome maintenance complex component 2 | -1.62 | -2.00 | -1.67 | 1.86 |
| PLK4 | polo-like kinase 4 | -1.57 | -1.90 | -2.17 | 2.01 |
| MCM6 | minichromosome maintenance complex component 6 | -1.57 | -1.34 | -1.43 | 1.48 |
| CDC2 | cyclin-dependent kinase 1 | -1.56 | -1.64 | -2.11 | 2.07 |
| MCM5 | minichromosome maintenance complex component 5 | -1.55 | -1.51 | -1.44 | 1.53 |
| CCNB1 | cyclin B1 | -1.54 | -1.45 | -1.95 | 1.81 |
| CCNB2 | cyclin B2 | -1.52 | -1.79 | -2.14 | 2.16 |
| E2F2 | E2F transcription factor 2 | -1.50 | -1.35 | -1.27 | 2.35 |
| AURKB | aurora kinase B | -1.50 | -1.89 | -2.03 | 2.94 |
| CKS2 | CDC28 protein kinase regulatory subunit 2 | -1.50 | -1.30 | -2.10 | 1.76 |

NR = No result
