# Supplementary material for: Regulation of p53 and Rb Links the Alternative NF-κB Pathway to EZH2 Expression and Cell Senescence
Source: PLoS Genet. 2014 Sep 25;10(9):e1004642. doi: 10.1371/journal.pgen.1004642 (PMC4177746; doi:10.1371/journal.pgen.1004642)
Supplement: Table S5 — Microarray gene expression data for RelB regulated genes associated with ubiquitin mediated degradation. (DOC) [file pgen.1004642.s015.doc]

Iannetti et al. Table S5.

RelB regulated genes associated with ubiquitin mediated degradation

| Gene Symbol | Gene Name | siNF-κB2 | siRelB | siEZH2 | sip53 |
| --- | --- | --- | --- | --- | --- |
| FBXO5 | F-box protein 5 | -1.64 | -1.75 | -1.79 | 2.23 |
| FKBP1A | FK506 binding protein 1A, 12kDa | NR | -1.58 | NR | NR |
| MAD2L1 | MAD2 mitotic arrest deficient-like 1 (yeast) | -1.61 | -1.87 | -2.17 | NR |
| ANAPC1 | anaphase promoting complex subunit 1 | NR | -1.72 | -1.20 | 1.30 |
| CDC16 | cell division cycle 16 homolog (S. cerevisiae) | NR | -1.58 | NR | -1.27 |
| CDC20 | cell division cycle 20 homolog (S. cerevisiae) | -1.87 | -1.80 | -2.67 | 1.79 |
| PSMA5 | proteasome (prosome, macropain) subunit, alpha type, 5 | NR | -1.51 | NR | -1.25 |
| TSC1 | tuberous sclerosis 1 | -1.23 | 1.53 | -1.26 | NR |
| UBE2C | ubiquitin-conjugating enzyme E2C | -1.64 | -1.72 | -2.20 | 2.24 |

NR = No result
