# Supplementary material for: Regulation of p53 and Rb Links the Alternative NF-κB Pathway to EZH2 Expression and Cell Senescence
Source: PLoS Genet. 2014 Sep 25;10(9):e1004642. doi: 10.1371/journal.pgen.1004642 (PMC4177746; doi:10.1371/journal.pgen.1004642)
Supplement: Text S1 — Details of oligonucleotides, siRNAs and PCR primer sequences. (DOC) [file pgen.1004642.s016.doc]

**Supporting Material: Oligonucleotide and siRNA information**

**siRNAs**

sip14^ARF^1: AACAUGGUGCGCAGGUUCUUG

sip14^ARF^2: AAGACCAGGUCAUGAUGAUGG

siRNA control: CAGUCGCGUUUGCGACUGG

siNF-κB2 (A): A pool of

(a) CAGCCUAAGCAGAGAGGCU

(b) CUACGAGGGACCAGCCAAG

(c) GAUGAAGAUUGAGCGGCCU

siNF-κB2(B): on-target plus siRNA (Dharmacon, J- 003918-05)

siRelB (A): UUGGAGAUCAUCGACGAGU

siRelB(B): on-target plus siRNA (Dharmacon, J-004767-08)

siBcl3: CAACCUACGGCAGACACCG

sip53 (A): GACUCCAGUGGUAAUCUAC

sip53 (B): on-target plus siRNA (Dharmacon, J-003329-14)

sip21^WAF1^: AGACCAGCAUGACAGAUUUCU

siEZH2(A): AAGACUCUGAAUGCAGUUGCU

siEZH2 (B): on-target plus siRNA (Dharmacon, J-004218-06)

siCDK4 (A): on-target plus siRNA (Dharmacon, J-003238-13)

siCDK4 (B): on-target plus siRNA (Dharmacon, J-003238-14)

siCDK6: on-target plus siRNA (Dharmacon, J-003240-12)

siDEK: on-target plus siRNA (Dharmacon, J-003881-09)

siRb: on-target plus SMARTpool siRNA (Dharmacon, J-003296),

siPSMA5 (A): on-target plus siRNA (Dharmacon J-011359-05)

siPSMA5 (B): on-target plus siRNA (Dharmacon, J-011359-07)

siANAPC1 (A): on-target plus siRNA (Dharmacon, J-013843-08)

siANAPC1 (B): on-target plus siRNA (Dharmacon, J-013843-10)

siMnSOD: on-target plus siRNA (Dharmacon, J-009784-05)

siRAC1: on-target plus siRNA (Dharmacon, J-003560-14)

siCdc42: on-target plus siRNA (Dharmacon, J-005057-05)

siRACGAP1: on-target plus siRNA (Dharmacon, J-008650-05)

siPUMA: on-target plus siRNA (Dharmacon, J-004380-09)

siUBE2C: on-target plus siRNA (Dharmacon, J-004693-09)

siCdc16: on-target plus siRNA (Dharmacon, J-003223-10)

siFbx05: on-target plus siRNA (Dharmacon, J-012434-08)

sitp53INP1: on-target plus siRNA (Dharmacon, J-016159-08)

**Q-PCR Oligonucleotides**

EZH2 FOR-GGGACAGTAAAAATGTGTCCTGC

EZH2 REV-TGCCAGCAATAGATGCTTTTTG

CDK4 FOR-AGAGTGTGAGAGTCCCCAATG

CDK4 REV-CAAACACCAGGGTTACCTTG

p53 FOR-TCAGACCTATGGAAACTACTT

p53 REV-GGGACAGCATCAAATCAT

NF-κB2 FOR-GGGCAGACCAGTGTCATTGAG

NF-κB2 REV-CCATGCCGATCCAGCAGAG

RelB FOR-CATCGAGCTCCGGGATTGT

RelB REV-CTTCAGGGACCCAGCGTTGTA

p16^INK4a^ FOR-GAAGGTCCCTCAGACATCCCC

p16^INK4a^ REV-CCCTGTAGGACCTTCGGTGAC

GAPDH FOR-GGTCGTATTGGGCGCCTGGTCACC

GAPDH REV-CACACCCATGACGAACATGGGGGC

p21: quantitect primer assay (Qiagen) cat. No. QT00062090

CDK6: quantitect primer assay (Qiagen) cat. No. QT00019985

DEK: quantitect primer assay (Qiagen) cat. No. QT 00073304

Tp53INP1: quantitect primer assay (Qiagen) cat. No. QT00233646

Bcl3: quantitect primer assay (Qiagen) cat. No. QT00008050

PSMA5: quantitect primer assay (Qiagen) cat. No. QT00071995

ANAPC1: quantitect primer assay (Qiagen) cat. No. QT00051884

CDC16: quantitect primer assay (Qiagen) cat. No. QT00060004

MnSOD: quantitect primer assay (Qiagen) cat. No. QT01008693

RACGAP1: quantitect primer assay (Qiagen) cat. No. QT00085953

p14ARF: quantitect primer assay (Qiagen) cat. No. QT00998452

Tp53INP1: quantitect primer assay (Qiagen) cat. No. QT00233646

**ChIP Primers**

Cdk4 promoter: -87/+108

CDK4 FOR: TCAAGCGGTCACGTGTGATA

CDK4 REV: ATGTGACCAGCTGCCAAAG

Cdk6 promoter: -455/-219

CDK6 FOR: GGTATTTACCACCCCCATTG

CDK6 REV: GAGAGAGTGGGACGTGTCGT

PSMA5 promoter: -87/+117

PSMA5 FOR: GTACCTCCCTGGTCCTCCTC

PSMA5 REV: GAGGACCAACACGACTCCAC

Anapc1 promoter: -248/-108

ANAPC1 FOR: CCCTGAGATCTATAAGGCAGT

ANAPC1 REV: GTTACAACTTGGGGGCTATT

Ezh2 promoter: +596/+888:

EZH2 FOR-GGACGGGACAGACACAAGTT

EZH2 REV-AAACGTCAGAGGCGAAGCTA

-2802/-2600 (Control Region):

FOR-ATCCTGAGCTCAAGCAGACC

REV-TCCAGGCCAGACTTAGTGGT
